# Disruption of the transcription factors Thi2p and Nrm1p alleviates the post-glucose effect on xylose utilization in *Saccharomyces cerevisiae*

**DOI:** 10.1186/s13068-018-1112-1

**Published:** 2018-04-16

**Authors:** Shan Wei, Yanan Liu, Meiling Wu, Tiantai Ma, Xiangzheng Bai, Jin Hou, Yu Shen, Xiaoming Bao

**Affiliations:** 1 State Key Laboratory of Microbial Technology, Microbiology and Biotechnology Institute, Shandong University, Shan Da Nan Road 27, Jinan, 250100 China; 2 School of Life Science, Shandong University, Shan Da Nan Road 27, Jinan, 250100 China; 3 Shandong Provincial Key Laboratory of Microbial Engineering, Qi Lu University of Technology, Daxue Rd 3501, Jinan, 250353 China

**Keywords:** *Saccharomyces cerevisiae*, Xylose metabolism, Post-glucose effect, *THI2*, *NRM1*, Bioethanol

## Abstract

**Background:**

The recombinant *Saccharomyces cerevisiae* strains that acquired the ability to utilize xylose through metabolic and evolutionary engineering exhibit good performance when xylose is the sole carbon source in the medium (designated the X stage in the present work). However, the xylose consumption rate of strains is generally low after glucose depletion during glucose–xylose co-fermentation, despite the presence of xylose in the medium (designated the GX stage in the present work). Glucose fermentation appears to reduce the capacity of these strains to “recognize” xylose during the GX stage, a phenomenon termed the post-glucose effect on xylose metabolism.

**Results:**

Two independent xylose-fermenting *S. cerevisiae* strains derived from a haploid laboratory strain and a diploid industrial strain were used in the present study. Their common characteristics were investigated to reveal the mechanism underlying the post-glucose effect and to develop methods to alleviate this effect. Both strains showed lower growth and specific xylose consumption rates during the GX stage than during the X stage. Glycolysis, the pentose phosphate pathway, and translation-related gene expression were reduced; meanwhile, genes in the tricarboxylic acid cycle and glyoxylic acid cycle demonstrated higher expression during the GX stage than during the X stage. The effects of 11 transcription factors (TFs) whose expression levels significantly differed between the GX and X stages in both strains were investigated. Knockout of *THI2* promoted ribosome synthesis, and the growth rate, specific xylose utilization rate, and specific ethanol production rate of the strain increased by 17.4, 26.8, and 32.4%, respectively, in the GX stage. Overexpression of the ribosome-related genes *RPL9A*, *RPL7B,* and *RPL7A* also enhanced xylose utilization in a corresponding manner. Furthermore, the overexpression of *NRM1*, which is related to the cell cycle, increased the growth rate by 8.7%, the xylose utilization rate by 30.0%, and the ethanol production rate by 76.6%.

**Conclusions:**

The TFs Thi2p and Nrm1p exerted unexpected effects on the post-glucose effect, enhancing ribosome synthesis and altering the cell cycle, respectively. The results of this study will aid in maintaining highly efficient xylose metabolism during glucose–xylose co-fermentation, which is utilized for lignocellulosic bioethanol production.

**Electronic supplementary material:**

The online version of this article (10.1186/s13068-018-1112-1) contains supplementary material, which is available to authorized users.

## Background

The production of biofuels and chemicals using lignocellulosic materials is a feasible strategy to meet future energy and resource needs. Xylose is the second most abundant sugar in hydrolysed lignocellulosic materials [1–4]. Therefore, the utilization of xylose in addition to glucose is a fundamental requirement of microorganisms for the conversion of bio-based fuels and chemicals. *Saccharomyces cerevisiae* is a robust and safe microorganism with a strong metabolism, and it is frequently used as a cell factory in the fermentation industry, particularly for ethanol production. Therefore, *S. cerevisiae* is considered the most promising microorganism that produces ethanol from lignocellulosic materials [5, 6]. However, *S. cerevisiae* lacks both an efficient xylose metabolic pathway and appropriate regulatory system to respond to xylose [7]. To build a xylose metabolic pathway in *S. cerevisiae* strains, heterologous xylose isomerase or xylose reductase and xylitol dehydrogenase were introduced into the strains [4, 8–10]. The genes for xylulokinase and the non-oxidative pentose phosphate pathway (PPP) were then overexpressed [3, 10–13]. The resultant strains demonstrated a basic capacity to convert xylose into ethanol via sequential xylulose-5-phosphate, PPP, and glycolysis steps [7]. Adaptive evolution was performed to further enhance xylose catabolism. The xylose conversion rate of these engineered strains significantly increased after a long cultivation time in medium with xylose as the sole carbon source [5, 7, 14–16].

To understand the elusive mechanisms underlying xylose fermentation, reverse metabolic engineering was carried out, and relevant factors were identified. Increased activity of the hexose transporter Hxt7 improved the absorption of xylose [17]. Deficiency of the aldose reductase Gre3 reduced the intracellular production of xylitol, which is an inhibitor of xylose isomerase, therefore enhancing xylose utilization [18]. A stress response regulator, Ask10, improved xylose isomerase activity by upregulating molecular chaperones, thereby enhancing xylose utilization [19]. Moreover, recently studies have shown that the use of carbon sources exerts substantial control over the metabolic status of *S. cerevisiae* [20, 21]. This was determined by investigating the glucose-sensing and repression network, which is composed of three signalling pathways [22, 23]. The Rgt2/Snf3–Rgt1 pathway primarily regulates the transcription of hexose transporters [24]; the Snf1–Mig1 pathway largely functions in repressing the genes involved in non-fermentable carbon metabolism [25]; and the most important pathway, the cAMP–PKA pathway, carries out genome-wide regulation by phosphorylating transcription factors (TFs) [22]. When glucose or another fermentable carbon source is present, cells maintain fermentative metabolism regardless of whether the conditions are aerobic or anaerobic [20, 21, 26, 27]. In this case, glycolysis and the PPP are activated in cells, while respiration and gluconeogenesis are repressed. Glucose is rapidly consumed and converted to ethanol. This phenomenon, which occurs during the fermentative phase of yeast growth, is called glucose repression [28]. When glucose becomes limited, the cells transiently arrest their growth and adjust their metabolism from fermentation to respiratory mode (a diauxic shift). Glycolysis and the PPP are suppressed, and respiration and gluconeogenesis are de-repressed [28]. The cells then restart their growth at a reduced rate by slowly consuming the ethanol that has accumulated in the medium. Salusjärvi et al. [29, 30] suggested that xylose is a semi-fermentable carbon source for *S. cerevisiae*, since the metabolism of yeast growing on xylose corresponds neither to that of fully glucose-repressed cells (fermentative state) nor to that of de-repressed cells (respiratory state), and the glucose signalling system plays an important role in xylose metabolism. Our previous work revealed that one of the glucose signalling pathways, Snf1/Mig1-mediated regulation, was reprogrammed in an evolved strain whose xylose consumption was enhanced [31]. Recently, the regulator Ira2, which is a negative regulator of Ras and an inhibitor of cAMP–PKA signalling [32], was also shown to affect xylose fermentation [7]. Moreover, unexpected control factors have been revealed through well-designed comparative transcriptome analysis. *ISU1* [encoding a scaffolding protein for mitochondrial iron–sulfur (Fe–S) cluster biogenesis] deficiency increased aerobic growth and xylose consumption rates, and the absence of *HOG1* [encoding a component of mitogen activated protein kinase (MAPK)] in the context of *isu1Δ* brought further improvement [7].

In contrast to the extensive efforts to improve xylose consumption and discover associated mechanisms, relatively little attention has been focused on the differing performances of strains undergoing glucose–xylose co-fermentation compare to strains engaging in fermentation using xylose as their sole carbon source [33]. Indeed, engineered *S. cerevisiae* strains ferment xylose at significantly lower rates during glucose–xylose co-fermentation compared to their good performance when xylose is the sole carbon source (referred to as the X stage in the present work) [1, 6, 34]. Moreover, during the xylose consumption phase after glucose is depleted in glucose–xylose co-fermentation, which is referred to as the GX stage in the present work, the growth and xylose consumption rates drop sharply rather than only decreasing to the levels observed during the X stage [1, 3, 6, 34, 35]. This phenomenon is referred to as the post-glucose effect on xylose metabolism. The post-glucose effect is generally observed in engineered xylose-utilizing *S. cerevisiae* strains with different genetic backgrounds, including the evolved strains mentioned above [1, 3, 6, 18, 34]. Transcriptome engineering [35] has focused on manipulating extant regulatory networks to enforce a state associated with a desired phenotype to improve xylose fermentation during the glucose–xylose co-utilization phase. Network models of well-known TFs have been used to guide such transcriptome engineering. Remarkably, deletions of *CAT8* (encoding a TF that is necessary for the de-repression of a variety of genes under non-fermentative growth conditions) and *HAP4* (encoding a transcriptional activator and global regulator of respiratory gene expression) reduced the carbon commitment to biomass during the glucose and xylose co-consumption phase, and the specific rate of ethanol production increased. However, no intervention has prevented the transition from fermentative to respiratory metabolism when cells enter the GX stage, and little is known about why cells show differing performances in xylose metabolism when glucose is present or not present in the medium.

To describe the mechanism underlying the post-glucose effect on xylose metabolism, two engineered xylose-utilizing *S. cerevisiae* strains, BSGX001 (haploid) and XH7 (diploid), were selected as test strains in the present work. BSGX001 was derived from the haploid strain CEN.PK113-5D, which has been widely used in metabolic engineering work [3, 12, 31, 36]. XH7 was derived from the diploid strain BSIF, which was isolated from a tropical fruit in Thailand [6, 37]. Both BSGX001 and XH7 express an exogenous xylose isomerase gene, overexpress *XKS1* and genes associated with the non-oxidative phase of the PPP, contain *GRE3* and *PHO13* deletions, and evolve in medium containing xylose as the sole carbon source. Transcriptome differences between the two strains were studied during the GX and X stages. The effects of disrupting all 11 TFs with differing levels of expression in the GX and X stages, as well as three metabolic genes, were also investigated. Our results revealed that *THI2* knockout and *NRM1* overexpression alleviated the post-glucose effect. Additional transcriptional and physiological work was performed, and the results suggest that these positive effects were related to the enhancement of ribosome synthesis and the cell cycle.

## Methods

### Plasmid and strain construction

The plasmids and *S. cerevisiae* strains used in this study are listed in Table 1. The primers used in this study are provided in Additional file 1: Table S1.

**Table 1:** *S. cerevisiae* strains and plasmids used in this study

*S. cerevisiae* strains and plasmids	Description	Sources
Plasmids		
pUG6	The plasmid with *LoxP*–*KanMX4*–*LoxP* cassette	[38]
pJX7	Yeast 2*μ* plasmid, *TEF1p*–*Ru*-*xylA*–*PGK1t, URA3* marker	[19]
YEp-CH	Shuttle plasmid for *E. coli* and *S. cerevisiae*, Cre gene under control of *GAL2* promoter, Hygromycin marker	Laboratory preserved
pIYC04	Yeast 2*μ* plasmid, *PGK1p*–*CYC1t*, *TEF1p*–*ADHt*, *HIS3* marker	[19]
pXIδ	Yeast 2*μ* plasmid, 3XI, δ1δ2, *KanMX4* marker	[6]
pUC20	Yeast 2*μ* plasmid, δ1δ2, *TEF1p*–*ADHt*, *KanMX4* marker	This study
pUC20–*FBA1*	pUC20, *TEF1p*–*FBA1*–*ADHt*	This study
pUC20–*TDH2*	pUC20, *TEF1p*–*TDH2*–*ADHt*	This study
pUC20–*GPM1*	pUC20 *TEF1p*–*GPM1*–*ADHt*	This study
pUC20-*RPL7A*	pUC20 *TEF1p*-*RPL7A* -*ADHt*	This study
pUC20–*RPL7B*	pUC20 *TEF1p*-*RPL7B*–*ADHt*	This study
pUC20–*RPL9A*	pUC20 *TEF1p*–*RPL9A–ADHt*	This study
pUC20–*RPL22A*	pUC20 *TEF1p*–*RPL22A* –*ADHt*	This study
pUC20–*RPL22B*	pUC20 *TEF1p*–*RPL22B*–*ADHt*	This study
*S. cerevisiae* strains		
CEN.PK 113-5D	*MATa; ura3*-*53*	[36]
XH7	Derived from a diploid *S. cerevisiae* strain isolated from tropical fruit in Thailand, *pho13*::XI, *gre3*::PPP, XK, 3δ::XI, AE^a^	[6]
BSGX001	CEN.PK 113-5D derivative; Ru-XI, XK, *gre3*::PPP, *cox4Δ*, AE^a^	[39]
BSGX001 (*aca1Δ*)^b^	BSGX001, *aca1::KanMX4*	This study
BSGX001 (*RPL7A*)^c^	BSGX001, δ1-*loxp*–*KanMX4*–*loxp*–*TEF1p*-*RPL7A*–*ADHt*-δ2	This study

^a^*AE* adaptive evolution in medium using xylose as the sole carbon source

^b^Other strains derived from BSGX001 with deleted genes were named in the same way, and because of lack of space they are not listed here

^c^Other strains derived from BSGX001 with overexpressed genes were named in the same way, and because of lack of space they are not listed here

A *TEF1p*–*ADH1t* fragment was amplified from plasmid pIYC04 with *Bam*H1 and *Sal*1 restriction sites at the 5′ and 3′ ends, respectively. The fragment was then ligated into plasmid pXIδ between the *Bam*H1 and *Sal*1 sites, resulting in pUC20. The ORFs of *FBA1, GPM1, TDH2, RPL7A, RPL7B, RPL9A, RPL22A,* and *RPL22B* were amplified from the genome of CEN.PK 113-5D with *Not*1 and *Pac*1 restriction sites at the 5′ and 3′ ends, respectively. These genes were then ligated into plasmid pUC20 between the *Not*1 and *Pac*1 sites, resulting in pUC20–*FBA1*, pUC20–*GPM1*, pUC20–*TDH2*, pUC20–*RPL7A,* pUC20–*RPL7B,* pUC20–*RPL9A,* pUC20–*RPL22A,* and pUC20–*RPL22B,* respectively (Table 1). All genes were expressed under control of the *TEF1* promoter. TF gene knockout was performed by homologous recombination using a *KanMX4* expression cassette to replace the target genes. Overexpression of TF genes was also performed by homologous recombination using the *TPI*1 promoter to replace the original promoter. All expression and deletion cassettes were verified by sequencing before transformation into BSGX001. The resulting strains are listed in Table 1. The *KanMX4* marker was then discarded by transferring plasmid YEp-CH into the strains and inducing the expression of Cre recombinase [12].

### Medium and growth conditions

*E. coli* recombinant cells were cultured at 37 °C in Luria–Bertani (LB) medium (5 g L^−1^ yeast extract, 10 g L^−1^ tryptone, 10 g L^−1^ NaCl, pH 7.0), and 100 mg L^−1^ ampicillin was added as necessary. Yeast cells were cultured at 30 °C in synthetic complete dropout uracil (SC-Ura) medium (1.7 g L^−1^ yeast nitrogen base, 5 g L^−1^ (NH_4_)_2_SO_4_, 0.77 g L^−1^ CSM-Ura (Sunrise Science Products, USA) supplemented with 20 g L^−1^ glucose as the carbon source. G418 (Promega Corporation, USA) (200 mg L^−1^ in liquid medium and 800 mg L^−1^ in solid medium) was added for transformant selection as necessary [40, 41]. The fermentation medium consisted of synthetic complete dropout (SC) medium with 20 g L^−1^ glucose and 20 g L^−1^ xylose or 20 g L^−1^ xylose alone as the carbon source.

### Fermentation

Overnight cultures of a single colony were transferred to fresh SC-Ura medium (50–60 mL) supplemented with 20 g L^−1^ glucose in 250-mL shake flasks at an initial OD_600_ of 1.0 and incubated at 30 °C and 200 rpm for 12–16 h. The cells were then collected and washed thrice with sterile water and resuspended in 1 mL of fermentation medium before inoculating into the fermentation medium. The initial biomass was 0.575 g L^−1^ dry cell weight (DCW; ~ 2.5 OD units). Fermentation was performed in shake flasks or 1-L bioreactors according to the experimental requirements. Fermentation in shake flasks was performed at 30 °C and 200 rpm. Fermentation in bioreactors was performed at 30 °C and pH 5.5, with 0.06-vvm air sparging and a stirring speed of 200 rpm. The pH was maintained by automatic pumping of 5 mol L^−1^ NaOH and 5 mol L^−1^ H_3_PO_4_. All fermentations were carried out in triplicate.

### Analysis of metabolites and calculations

Fermentation samples were collected at specific time intervals. The cell density (OD_600_) was determined with a UV–visible spectrophotometer (Eppendorf, Germany). Calculation of the DCW followed a previously described method [12]. One OD_600_ unit corresponded to 0.230 g of DCW L^−1^ for BSGX001 and its derivative strains [12] and 0.188 g DCW L^−1^ for XH7. The concentrations of glucose, xylose, glycerol, acetate, and ethanol were determined using HPLC (Shimadzu, Japan) with an Aminex HPX-87H ion exchange column (300 × 7.8 mm) (Bio-Rad, Hercules, USA). H_2_SO_4_ (5 mmol L^−1^) was used as the mobile phase with a flow rate of 0.6 mL min^−1^, and the temperature of the column oven was 45 °C [6, 12]. The specific growth rate (*μ*) was the regression coefficient of the log-linear regression of the OD_600_ versus time during the exponential growth phase [42]. The specific xylose utilization rate (*r*_xylose_) and specific ethanol production rate (*r*_ethanol_) were calculated using the following equation, as previously described [12]:

$$r = \frac{{A_{n} - A_{m} }}{{\frac{1}{2}\sum\nolimits_{i = m + 1}^{n} {(B_{i} + B_{i - 1} ) \times (t_{i} - t_{i - 1} )} }},$$

where *r* is the specific utilization or production rate during the phase from sampling point *m* to sampling point *n*, and *A*, *B*, and *t* are the metabolite concentration, biomass concentration, and time, respectively, at sampling points *n*, *i*, and *m*.

### Transcriptome analysis

RNA-seq was carried out for the transcriptome analysis. Samples were taken from the duplicate batch fermentations in the bioreactors. The samples taken at 14 h from the glucose–xylose co-fermentation were defined as GX stage samples. The samples taken at 12 h from the fermentation using xylose as the sole carbon source were defined as X stage samples. The cells in each sample were collected by centrifugation at 5000 rpm and 4 °C for 5 min. The resulting pellets were rapidly frozen in liquid nitrogen and stored at − 80 °C until RNA extraction was performed [19].

Total RNA was extracted using a UNIQ-10 Trizol RNA Purification Kit (Sangon Biotech, China). Total RNA was extracted and fragmented, DNA was digested with DNase I, and cDNA was synthesized by using short mRNA fragments as templates. The short fragments were connected with a connector, suitable fragments were selected, and then PCR amplification was performed. Finally, the sample library was sequenced using an Illumina HiSeq™ 2000 (performed by Beijing Genomics Institute).

The raw data from the transcriptional analysis and the processed data for genes exhibiting significant differences between the GX and X stages are available in the NCBI Gene Expression Omnibus database (GEO Accession Number: GSE95076). Significant differences were indicated by *p* values of 0.001 or less and an absolute fold-change threshold of 2.0 or greater. The probability deviation value [43] was 0.80 or greater. All annotations were derived from the Saccharomyces Genome Database (SGD) (http://www.yeastgenome.org/). Cluster analysis was also performed using the tools supplied by the SGD.

### Quantitative PCR (qPCR)

qPCR samples were taken from batch fermentations in shake flasks containing the fermentation medium. GX stage samples of both strains were taken at 14 h during glucose–xylose co-fermentation. X stage samples of both strains were taken at 12 h during fermentations with xylose as the sole carbon source. *ACT1* was used as the reference gene. The real-time qPCR data were analysed according to the 2^−ΔΔCT^ method [44].

### Cell cycle analysis

Cells were activated three times and then transferred into fresh fermentation medium with an initial OD_600_ of 2.5. The activation was performed by culturing cells in SC-Ura medium supplemented with 20 g L^−1^ glucose for 12–16 h. Samples were taken every 2 h for separate triplicate tests and were analysed by flow cytometry on a Becton–Dickinson FACScan with Sytox [45].

## Results

### The metabolic activities of strains in the GX stage were much lower than those in the X stage

To describe the mechanism underlying the post-glucose effect on xylose metabolism, two engineered xylose-utilizing *S. cerevisiae* strains with different genetic backgrounds, BSGX001 and XH7, were selected as test strains. Fermentation was performed in 1-L bioreactors using SC medium supplied with 20 g L^−1^ glucose and 20 g L^−1^ xylose or 20 g L^−1^ xylose alone. The initial biomass was 0.575 g L^−1^ DCW (~ 2.5 OD units). The fermentation characteristics are shown in Table 2 and Additional file 1: Figure S1. For BSGX001, the specific growth rate, xylose consumption rate, and ethanol production rate during the GX stage were 78.5, 30.4, and 48.1% lower than those calculated for the X stage, respectively. For XH7, the specific growth rate was below detection limits during the GX stage, and the xylose consumption rate and ethanol production rate during the GX stage were 58.6 and 63.8% lower than those calculated for the X stage, respectively. These data indicate the much lower metabolism of strains in the GX stage than the X stage.

**Table 2:** Fermentation characteristics of two xylose utilizing strains

Strains	Fermentation stage	*μ* ^a^	*r*_xylose_^b^ (g g^−1^ DCW h^−1^)	*r*_ethanol_^b^ (g g^−1^ DCW h^−1^)
BSGX001	X	0.107 ± 0.002	0.461 ± 0.003	0.214 ± 0.002
	GX	0.023 ± 0.000*	0.321 ± 0.010*	0.111 ± 0.002*
XH7	X	0.103 ± 0.000	0.747 ± 0.002	0.340 ± 0.004
	GX	–^c^	0.309 ± 0.000*	0.123 ± 0.004*

Fermentation in bioreactors was performed at 30 °C and pH 5.5, with 0.06-vvm air sparging and a stirring speed of 200 rpm. All the data are the mean value ± standard deviation of independent triplicate tests

* *p* < 0.05

^a^The specific growth rates (*μ*) were calculated from the data on the xylose-only consumption phase in glucose–xylose co-fermentation (GX stage) and exponential growth phase in xylose fermentation (X stage)

^b^The specific consumption/production rates of xylose/ethanol (*r*_xylose_/*r*_ethanol_) were calculated from the data on the xylose-only consumption phase in glucose–xylose co-fermentation (GX stage) and exponential growth phase in xylose fermentation (X stage)

^c^Below detection limits

### Transcriptional differences between cells in the GX stage and X stage

**Fig. 1:**
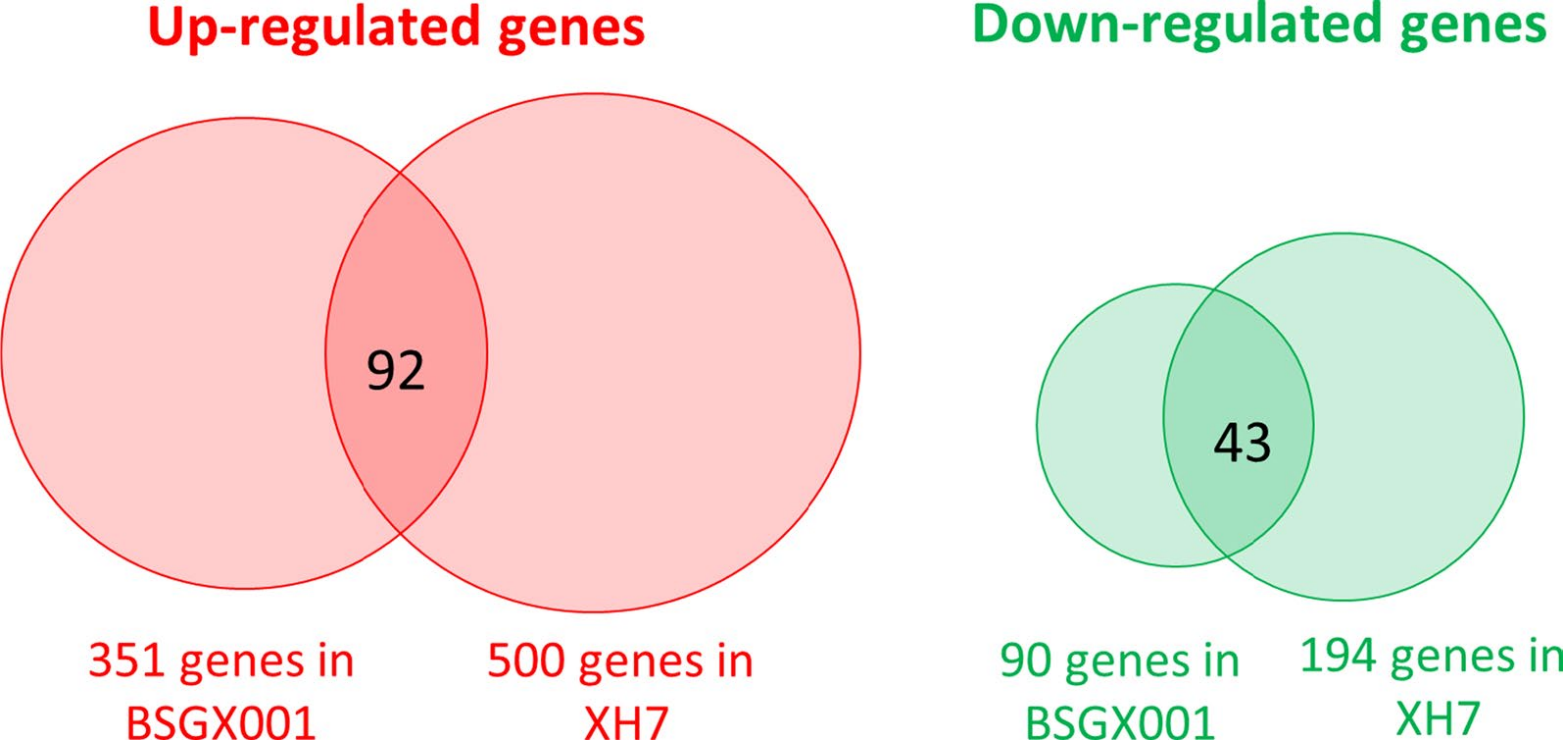
The number of significantly regulated genes in BSGX001 and XH7 (GX stage vs X stage). Transcriptome analysis was performed on both BSGX001 and XH7. GX stage samples were taken at 14 h (2 h after glucose depletion) from glucose–xylose co-fermentation. X stage samples were taken at 12 h (the middle of the exponential phase) from xylose fermentation. Respectively, 92 and 43 genes were up- and down-regulated in both strains

Transcriptome analysis was performed on both BSGX001 and XH7. The GX stage samples were taken at 14 h (2 h after glucose depletion) during glucose–xylose co-fermentations (Additional file 1: Figure S1). At that time, ~ 12–13 g L^−1^ xylose remained in the medium. The X stage samples were taken at 12 h (the middle of the exponential phase) during fermentations with xylose as the sole carbon source. At that time, ~ 8–10 g L^−1^ xylose residue remained in the medium (Additional file 1: Figure S1). The results revealed that 351 and 500 genes were up-regulated in BSGX001 and XH7, respectively, and 90 and 194 genes were down-regulated in BSGX001 and XH7, respectively. The intersection of up- and down-regulated genes in these two strains included 92 and 43 genes, respectively (Fig. 1). These overlapping genes were clustered according to Gene Ontology (GO) terms by using the Gene Ontology Slim Mapper tool supplied by the Saccharomyces Genome Database (http://www.yeastgenome.org). The results (Additional file 1: Table S2) revealed that within the molecular functions category, the up-regulated genes were primarily clustered (cluster frequency ≥ 10%) under the GO terms of transmembrane transporter activity and hydrolase activity; the down-regulated genes were primarily clustered under the GO terms of structural constituents of the ribosome, transferase activity, and oxidoreductase activity. Within the biological processes category, the up-regulated genes were primarily clustered under the GO terms of responses to chemical and ion transport; the down-regulated genes were primarily clustered under the GO terms of cellular amino acid metabolic processes, cytoplasmic translation, and rRNA processing.

**Fig. 2:**
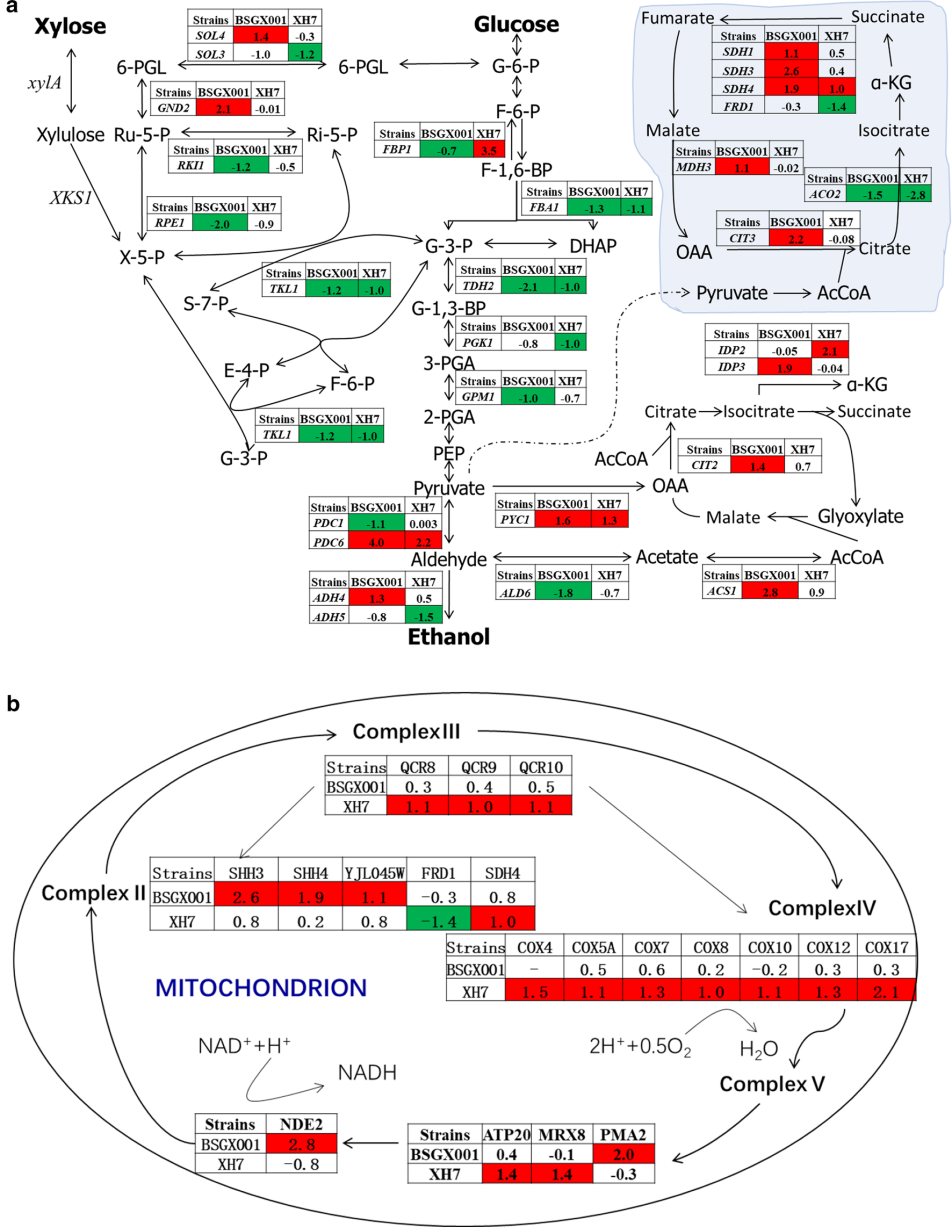
The expression difference of metabolic genes between the GX stage and X stage in BSGX001 and XH7. **a** Glycolysis, the pentose phosphate pathway, tricarboxylic acid cycle, and glyoxylic acid cycle; **b** mitochondrial function genes, including the electron transport chain and oxidative phosphorylation. The data are presented as the log_2_ (fold change) of genes. Red and green represent up- and down-regulation, respectively

Changes in central carbon metabolic pathways were also analysed. The expression levels of genes involved in glycolysis and the PPP were lower during the GX stage than during the X stage for both strains (Fig. 2a). Genes involved in the tricarboxylic acid cycle and glyoxylic acid cycle (Fig. 2a), as well as genes in the electron transport chain and ATP biosynthesis in the mitochondria (Fig. 2b), showed higher expression levels during the GX stage than during the X stage. This physiological reaction resembled the general physiological reaction observed for *S. cerevisiae* under glucose depletion [28]. In addition, some glucose-repressed genes, such as genes involved in fructose, mannose, galactose, sucrose, and starch metabolism [24], were also expressed at higher levels in the GX stage than in the X stage in both strains (Additional file 1: Figure S2). This indicated that these genes are repressed during the X stage, but are de-repressed during the GX stage. Moreover, within our transcriptome analysis, 32.6% of down-regulated genes in the GX stage compared to the X stage were clustered under the GO term of cellular amino acid metabolic processes (Additional file 1: Table S2).

### The expression levels of the glycolysis genes *FBA1*, *GPM1*, and *TDH2* are not bottlenecks during xylose fermentation

In *S. cerevisiae*, xylose is sequentially metabolized through the PPP and glycolysis and is then converted to end products such as ethanol (Fig. 2a). Therefore, maintaining highly active PPP and glycolysis is important for achieving high xylose fermentation efficiency [46]. Although the importance of PPP in xylose metabolism has been extensively studied [6, 39], less attention has been given to glycolysis. Therefore, we compared the expression levels of glycolysis genes between the GX and X stages in the two xylose-utilizing strains. The expression of many genes was changed; among these, *FBA1* (encoding fructose 1,6-bisphosphate aldolase)*, GPM1* (encoding phosphoglycerate mutase), and *TDH2* (encoding glyceraldehyde-3-phosphate dehydrogenase isozyme 2) were significantly decreased in both strains (Fig. 2a). Therefore, their roles in the post-glucose effect were investigated.

To verify whether the decreased expression of these genes directly affected the efficiency of xylose metabolism, *FBA1*, *GPM1*, and *TDH2* were overexpressed in BSGX001. The fermentation performance of the strains was evaluated in shake flasks. The results showed that the overexpression of *FBA1* and *GPM1* did not enhance xylose fermentation and that overexpressed *TDH2* completely blocked the utilization of xylose after glucose depletion (Table 3).

**Table 3:** The xylose fermentation characteristics of strain overexpressing *FBA1*, *GPM1*, or *TDH2*

Genotype of strains	*μ* ^a^	*r*_xycose_^b^ (g g^−1^ DCW h^−1^)	*r*_ethanol_^b^ (g g^−1^ DCW h^−1^)
BSGX001	0.162 ± 0.001	0.065 ± 0.002	0.033 ± 0.008
*FBA1*	0.164 ± 0.002*	0.060 ± 0.001*	0.032 ± 0.002
*GPM1*	0.158 ± 0.001*	0.056 ± 0.006	0.031 ± 0.008
*TDH2*	0.160 ± 0.001*	0.000 ± 0.000*	− 0.013 ± 0.000*

Cells were cultured at 30 °C in a shake flask and agitated at 200 rpm. All the data are the mean value ± standard deviation of independent triplicate tests

* *p* < 0.05

^a^The specific growth rate (μ) was calculated from the data on the glucose consumption phase in glucose–xylose co-fermentation

^b^The specific consumption/production rates of xylose/ethanol (*r*_xylose_/*r*_ethanol_) were calculated from the data on the xylose-only consumption phase in the glucose–xylose co-fermentation (GX stage)

### Effects of disrupting TFs whose expression levels significantly differed between the GX stage and X stage

**Fig. 3:**
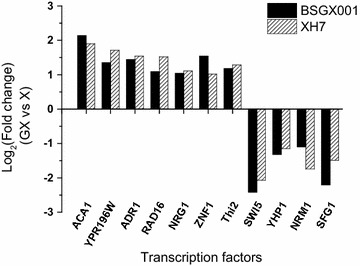
TFs with significantly different expression levels in the GX stage versus the X stage in both BSGX001 and XH7

Previous studies [7, 19, 31] describing the mechanisms underlying xylose metabolism (reviewed above in the introduction) and our transcriptome analysis results all indicate that the post-glucose effect on xylose metabolism is complex and related to network regulation. It is well known that TFs are widely distributed in the regulatory networks of genes for diverse biological processes [47, 48]. The disruption of TFs to induce a state that is associated with a desired phenotype or to investigate a gene-regulatory network has previously been broadly applied [35, 47, 49]. It is possible that TFs whose expression differed significantly between the GX stage and X stage in both strains are involved in the post-glucose effect. *ACA1*, *ADR1*, *NRG1*, *RAD16*, *YPR196W*, and *ZNF1*, which were classified as TF genes (Additional file 1: Table S2), exhibited significantly different expression (Log_2_ (fold change) ≥ 1 or ≤ − 1, probability ≥ 0.80) between the GX stage and X stage in both BSGX001 and XH7. The TF gene *SFG1*, which was not classified under a particular GO term after analysis, was also differentially expressed; and *NRM1, THI2,* and *YHP1* had slightly lower probabilities (Log_2_ (fold change) ≥ 1 or ≤ − 1, probability ≥ 0.76). Among these 11 significantly differentially expressed TFs in both strains (Fig. 3), 7 demonstrated increased expression in the GX stage, and 4 of these are involved in carbon metabolism: Adr1p is an activator of respiratory metabolism genes [50]; Znf1p activates genes involved in respiration, gluconeogenesis, and the glyoxylate shunt [51]; Nrg1p is a repressor that mediates glucose repression [52]; and Aca1p belongs to the ATF/CREB family and is also important for carbon source utilization [53]. The other three TFs do not have a clear connection to central carbon metabolism: Rad16p binds damaged DNA during global genome nucleotide-excision repair [54], Ypr196wp is a putative maltose-responsive TF [55], and Thi2p is an activator of thiamine biosynthetic genes [56]. Of the 4 TFs with decreased expression in the GX stage, all are related to the cell cycle. Nrm1p is a transcriptional co-repressor of MBF-regulated gene expression, which represses transcription upon exit from the G1 phase [50, 57]. Yhp1p is a transcriptional repressor that restricts the G1/S transition in the mitotic cell cycle [58]. Swi5p activates the transcription of genes expressed at the M/G1 phase boundary and in the G1 phase [59]. Sfg1p mediates nutrient-dependent regulation of ribosome biogenesis and cell size [60]. These genes were individually overexpressed and deleted in BSGX001 to study their effects on fermentation characteristics. The data obtained from shake flask fermentations (Table 4) revealed that, in general, disruption of these TFs did not have a positive effect on xylose utilization, with the exception of *THI2* knockout and *NRM1* and *YHP1* overexpression. Knocking out *ZNF1, NRG1,* and *YPR196W* or overexpressing *SWI5* and *SFG1* strongly decreased xylose utilization.

**Table 4:** The fermentation characteristics of strains knocking out or overexpressing TFs whose expression levels were significantly different between the GX stage and X stage

Genotype of strains	Glucose–xylose co-fermentation	Xylose fermentation		
	*r*_xylose_^a^ (g g^−1^ DCW h^−1^)	*μ* ^b^	*r*_xylose_^c^ (g g^−1^ DCW h^−1^)	*r*_ethanol_^c^ (g g^−1^ DCW h^−1^)
Control (BSGX001)	0.065 ± 0.002	0.082 ± 0.002	0.645 ± 0.001	0.216 ± 0.002
Positive operation^d^				
*adr1Δ*	0.046 ± 0.002*	0.075 ± 0.001*	0.642 ± 0.001*	0.235 ± 0.001*
*aca1Δ*	0.054 ± 0.001*	0.083 ± 0.002*	0.667 ± 0.010	0.212 ± 0.001*
*znf1Δ*	0.048 ± 0.001*	0.070 ± 0.001*	0.531 ± 0.008*	0.226 ± 0.002*
*nrg1Δ*	0.023 ± 0.000*	0.067 ± 0.001*	0.488 ± 0.015	0.166 ± 0.003*
*rad16Δ*	0.051 ± 0.001*	0.080 ± 0.002*	0.600 ± 0.002	0.244 ± 0.005*
*ypr196wΔ*	0.039 ± 0.000*	0.080 ± 0.002*	0.650 ± 0.002*	0.257 ± 0.004*
*thi2Δ*	0.109 ± 0.002*	0.069 ± 0.003*	0.505 ± 0.000*	0.206 ± 0.001*
*SWI5*	0.038 ± 0.001*	0.078 ± 0.003*	0.610 ± 0.010	0.220 ± 0.001*
*SFG1*	0.037 ± 0.002*	0.069 ± 0.000*	0.509 ± 0.001*	0.179 ± 0.002*
*YHP1*	0.040 ± 0.001*	0.103 ± 0.002*	0.660 ± 0.001*	0.226 ± 0.001*
*NRM1*	0.088 ± 0.001*	0.097 ± 0.001*	0.710 ± 0.002*	0.253 ± 0.001*
Negative operation^d^				
*ADR1*	0.013 ± 0.003*	0.082 ± 0.002*	0.664 ± 0.001*	0.173 ± 0.002*
*ACA1*	0.051 ± 0.001*	0.080 ± 0.001*	0.600 ± 0.002*	0.200 ± 0.002*
*ZNF1*	0.045 ± 0.001*	0.077 ± 0.002*	0.630 ± 0.002*	0.210 ± 0.001*
*NRG1*	0.041 ± 0.000*	0.070 ± 0.003*	0.521 ± 0.002*	0.189 ± 0.003*
*RAD16*	0.005 ± 0.001*	0.067 ± 0.002*	0.489 ± 0.001*	0.186 ± 0.005*
*YPR196W*	0.031 ± 0.001*	0.080 ± 0.002*	0.650 ± 0.002*	0.257 ± 0.002*
*THI2*	0.049 ± 0.000*	0.067 ± 0.000*	0.487 ± 0.002*	0.157 ± 0.000*
*swi5Δ*	0.048 ± 0.002*	0.078 ± 0.002*	0.638 ± 0.004*	0.266 ± 0.001*
*sfg1Δ*	0.053 ± 0.002*	0.066 ± 0.000*	0.487 ± 0.003*	0.169 ± 0.001*
*yhp1Δ*	0.034 ± 0.002*	0.080 ± 0.001*	0.600 ± 0.002*	0.215 ± 0.001*
*nrm1Δ*	0.015 ± 0.000*	0.080 ± 0.002*	0.637 ± 0.002*	0.266 ± 0.002*

Cells were cultured at 30 °C in a shake flask and agitated at 200 rpm. All the data are the mean value ± standard deviation of independent triplicate tests

* *p* < 0.05

^a^*r*_xylose_/*r*_ethanol_ was calculated from data on the xylose-only consumption phase in glucose–xylose co-fermentation (GX stage)

^b^*μ* was calculated from data on the exponential growth phase of xylose fermentation (X stage)

^c^The specific consumption/production rates of xylose/ethanol (*r*_xylose_/*r*_ethanol_) were calculated from data on X stage

^d^The positive operation represents overexpressed genes with lower expression in the GX stage compared to the X stage or knocked out genes with higher expression in the GX stage compared to the X stage; the negative operation represents the reverse operation

### Deletion of the TF gene *THI2* alleviates the post-glucose effect by enhancing ribosome synthesis

The shake flask fermentation results revealed that knocking out *THI2* increased the specific xylose utilization rate by 67.7% in the GX stage (Table 4). Conversely, overexpressing *THI2* decreased the specific xylose utilization rate by 24.6% (Table 4). This result is similar to that obtained in a previous study, in which overexpressing *THI2* repressed cellobiose fermentation [49]. In contrast, both deletion and overexpression of *THI2* had a negative effect on xylose utilization in the X stage. Therefore, knocking out *THI2* specifically enhanced xylose utilization in the GX stage, but not in the X stage (Table 4). This result was also replicated in fermentations performed in bioreactors, where conditions were strictly controlled. The specific growth rate, specific xylose utilization rate, and specific ethanol production rate of the *THI2* deletion strains were 17.4, 26.8, and 32.4% higher than the control, respectively (Table 5).

**Table 5:** The xylose fermentation characteristics of strain knocking out *THI2* and overexpressing *NRM1*

Genotype of strains	*μ* ^a^	*r*_xylose_^b^ (g g^−1^ DCW h^−1^)	*r*_ethanol_^b^ (g g^−1^ DCW h^−1^)
BSGX001	0.023 ± 0.000	0.321 ± 0.010	0.111 ± 0.002
BSGX001 (*thi2*)	0.027 ± 0.002*	0.407 ± 0.010*	0.147 ± 0.010*
BSGX001 (*NRM1*)	0.025 ± 0.000*	0.417 ± 0.020*	0.196 ± 0.000*

Cells were cultured in bioreactors at 30 °C and pH 5.5, with 0.06 vvm air sparging and a stirring speed of 200 rpm. All the data are the mean value ± standard deviation of independent triplicate tests

* *p* < 0.05

^a^The specific growth rate (*μ*) was calculated from the data on the xylose-only consumption phase in glucose–xylose co-fermentation (GX stage)

^b^The specific consumption/production rates of xylose/ethanol (*r*_xylose_/*r*_ethanol_) were calculated from the data on the GX stage

**Fig. 4:**
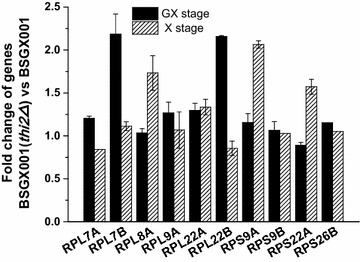
The fold change of RPs genes when deleting *THI2* in the GX and X stage. All the data for these samples are triplicate tests. Error bar standard deviation of three replicates

To investigate how *THI2* knockout enhanced xylose utilization, we re-analysed the transcriptome data of a *THI2* deletion strain generated in a previous study investigating the transcriptomes of deficiency mutants for the majority of TFs in *S. cerevisiae* [61]. Cluster analysis results revealed that 37.5% of up-regulated genes in the *THI2* deletion strain were ribosomal protein (RP) genes (Additional file 1: Table S3). Correspondingly, our transcriptome data showed that ribosomal-related genes were down-regulated in the GX stage compared to the X stage in both BSGX001 and XH7 (Additional file 1: Tables S2, S4). This result suggests that xylose metabolism might be related to ribosome synthesis, and *THI2* deletion might enhance xylose fermentation by enhancing ribosome synthesis. For verification, RP genes whose expression levels in the GX stage were notably lower than those in the X stage in both BSGX001 and XH7 were selected for investigation (Additional file 1: Table S4), and their expression levels in BSGX001 and BSGX001 (*thi2Δ*) were determined by qPCR. The results demonstrated that knocking out *THI2* increased the expression of ribosome-related genes (Fig. 4). The mRNA levels of *RPL7B* and *RPL22B* were increased in the GX stage after knocking out *THI2*, and the expression of *RPL7A, RPL9A,* and, *RPL22A* was also slightly enhanced in the GX stage (Fig. 4). The fermentation of strains overexpressing *RPL7A*, *RPL7B*, *RPL9A*, *RPL22A*, and *RPL22B* was then tested. The results (Table 6) showed that overexpressing *RPL22A* and *RPL22B* did not enhance xylose utilization; however, overexpression of *RPL9A*, *RPL7B,* and *RPL7A* did increase the specific xylose utilization rate by 21.3, 7.5, and 6.3%, respectively. Therefore, increasing the expression level of certain ribosomal proteins was beneficial to xylose utilization.

**Table 6:** The effect of overexpressing the RPs whose mRNA levels were increased in *THI2* deletion strains on xylose metabolism

Genotype of strains	*r*_xylose_^a^ (g g^−1^ DCW h^−1^)
BSGX001	0.080 ± 0.002
*RPL7A*	0.085 ± 0.001*
*RPL7B*	0.086 ± 0.001*
*RPL9A*	0.097 ± 0.001*
*RPL22A*	0.029 ± 0.000*
*RPL22B*	0.068 ± 0.000*

Cells were cultured at 30 °C in a shake flask and agitated at 200 rpm. All the data are the mean value ± standard deviation of independent triplicate tests

* p < 0.05

^a^The specific consumption rates of xylose (*r*_xylose_) were calculated from the data on the xylose-only consumption phase in glucose–xylose co-fermentation (GX stage)

### Overexpression of the cell cycle-related TF gene *NRM1* enhances xylose fermentation

Overexpression of *NRM1* increased the specific xylose utilization rate by 35.4% in the GX stage (Table 4). Conversely, deleting *NRM1* decreased the specific xylose utilization rate by 76.9%. This result was also observed in bioreactor fermentations. The specific growth rate, specific xylose utilization rate, and specific ethanol production rate of strains overexpressing *NRM1* were 8.7, 30.0, and 76.6% higher than the control, respectively (Table 5). Furthermore, overexpressing *NRM1* also enhanced cell growth and xylose utilization in the X stage (Table 4). The specific xylose utilization rate and the specific ethanol production rate increased by 10.1 and 17.1%, respectively. These results demonstrate that *NRM1* overexpression benefits xylose utilization in both the GX stage and the X stage.

Although *YHP1* overexpression did not significantly change the specific xylose consumption rate of the strains in either stage (Table 4), it enhanced the cell growth and xylose utilization in the X stage (Table 4): the specific growth rate increased by 25.6%, and the volumetric xylose utilization rate increased by 5.6%.

**Fig. 5:**
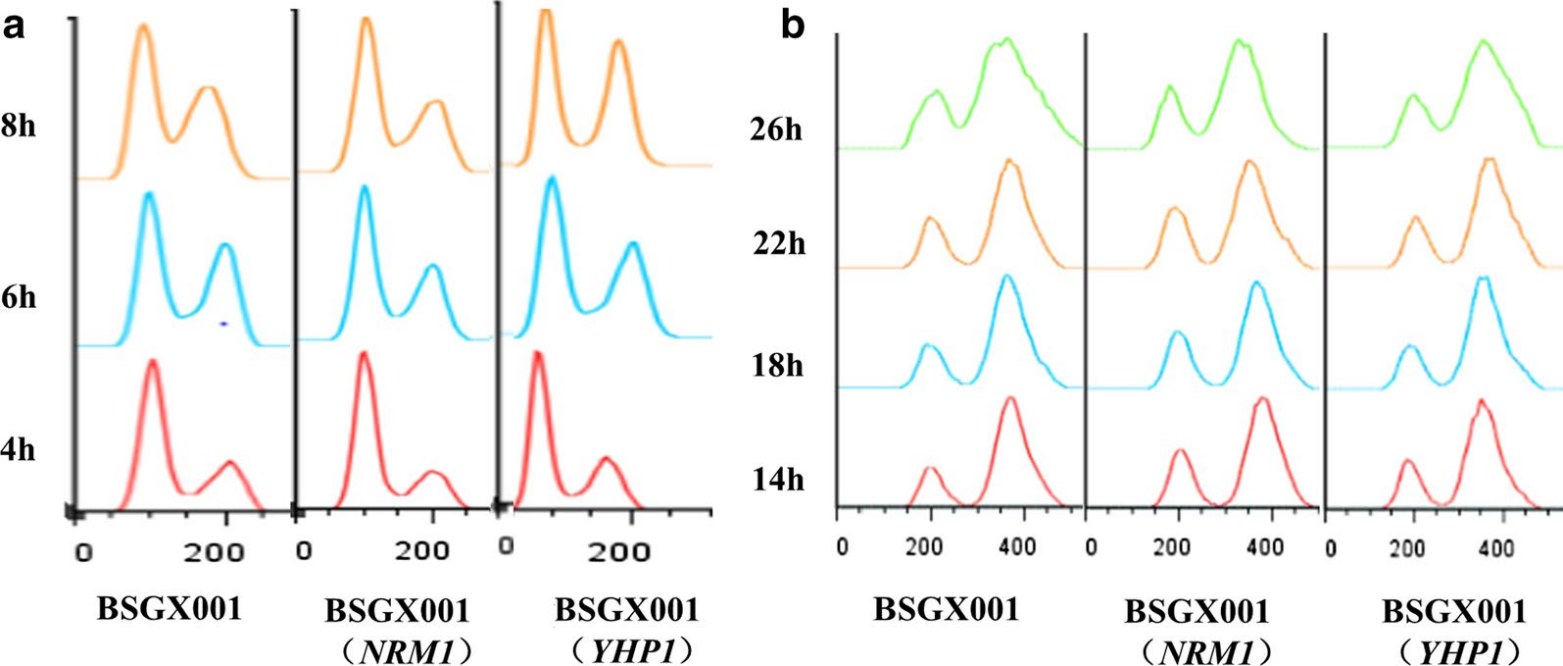
Effects of overexpression of *NRM1* and *YHP1* on the cell cycle. **a** Cells in the X stage. **b** Cells in the GX stage. Samples were subjected to independent triplicate tests

*NRM1* and *YHP1* are both related to the cell cycle. The FACS results (Fig. 5) indicated that cells were mainly found in the G2 phase during the GX stage, and there were no obvious differences between strains overexpressing *NRM1* or *YHP1* and the control, BSGX001. In the X stage, changes in the peak shape suggested changes in the cell cycle. When combined with the observed changes in the growth rate, it is possible that the cell cycles of the *NRM1* and *YHP1* overexpression strains accelerated. However, the details of this acceleration and how the cell cycle affects xylose metabolism are not yet clear.

## Discussion

When microorganisms are used for the production of bio-based fuels and chemicals from lignocellulosic materials, it is necessary that they possess the capacity to ferment xylose as well as glucose. In recent decades, the xylose metabolism of *S. cerevisiae* recombinant strains has greatly improved through metabolic and evolutionary engineering [5, 7, 14–16]. However, little effort has been made to understand why cells in the xylose consumption phase of glucose–xylose co-fermentation (the GX stage) exhibit significantly lower metabolic activity than cells undergoing fermentation with xylose as the sole carbon source (the X stage) and, more importantly, how to overcome this problem. It appears that xylose metabolism is prevented by glucose, even when glucose has been exhausted by the cells; therefore, we describe this phenomenon as the post-glucose effect on xylose metabolism.

To reveal the common features of strains that show a post-glucose effect on xylose metabolism, two xylose-utilizing strains with different genetic backgrounds were selected for study to clarify and narrow down potentially relevant targets. The transcriptional analysis results showed that the strains exhibited low-level glycolysis and a de-repressed tricarboxylic acid cycle. In terms of metabolism, both strains underwent fermentative metabolism, which resembled glucose repression, in the X stage. Meanwhile, growth and the specific xylose consumption rate (the xylose consumption rate per unit biomass) were much lower in the GX stage, very similar to cells in the lag phase. The transcriptional analysis results also indicated that these strains maintained a glucose repression state in the X stage, but shifted to a glucose de-repressed state in the GX stage. Several recent studies reported that recombinant *S. cerevisiae* exhibits carbon starvation during xylose fermentation [2, 8, 62]. Moreover, carbon starvation traits depend on the xylose consumption rate. Strains that have a high xylose utilization capacity display few of these traits [8]. Both recombinant strains we chose have a high xylose utilization capacity and ferment xylose well in the X stage. Accordingly, these strains did not exhibit starvation in the X stage. It is not surprising, however, that carbon starvation traits such as low ribosomal biogenesis and cell cycle arrest were observed in the GX stage, as these traits are highly linked to a low rate of sugar metabolism [63].

Previous work has often suggested that alterations in metabolic status depend on how glucose-sensing systems respond to the carbon source and how this response impacts the rate of glucose uptake [7, 20, 21, 31, 64]. Strains with modified metabolic pathways utilize xylose only at a very slow rate, primarily through respiratory metabolism. After strain evolution on xylose, the xylose consumption rate significantly increases and exhibits characteristics of fermentative metabolism when xylose is the sole carbon source (in the X stage), similar to our observations. Therefore, the glucose-sensing systems of the test strains in the present study were likely reprogrammed during evolution to enable them to recognize xylose as a fermentative (or semi-fermentative) carbon source and to enable fermentative metabolism in the X stage. This response was not as strong as the response to glucose, since the consumption rate of xylose remained lower than that of glucose. Based on these observations, the growth and metabolism of the strains were predicted to decrease to levels similar to those in the X stage after the depletion of glucose during glucose–xylose co-fermentation. However, this is not what occurred; instead, growth and metabolism were much lower than those observed in the X stage, similar to cells failing to recognize xylose during glucose–xylose co-fermentation and inducing carbon starvation [2, 8, 62] after the glucose was depleted. Of interest were the differing behaviours of cells during the GX and X stages. Both strains were unable to maintain growth and xylose consumption during the GX stage, unlike the X stage. In other words, we endeavoured to understand why cells undergoing glucose–xylose co-fermentation failed to recognize xylose after glucose was depleted. The transcription of all hexose transporters was similar in the X and GX stages, indicating that the differences in growth and metabolism were not caused by xylose transport. Moreover, since xylose is recognized by the Snf3p/Rgt2p pathway and regulates the expression of *HXT2*-*3* [29], similar expression levels of *HXT2*-*3* in the X and GX stages indicate that the Snf3p/Rgt2p pathway responses did not differ between the X and GX stages. Compared to the Snf3p/Rgt2p pathway, the other two pathways for glucose-sensing systems are much more complex. However, the expression of genes involved in respiration, gluconeogenesis, and the metabolism of alternative carbon sources, which are mainly regulated by the Snf1–Mig1 pathway [65], were low during the X stage but high during the GX stage, confirming that the Snf1–Mig1 pathway was active in the X stage, but not in the GX stage. Similarly, the high and low levels of glycolysis and growth observed in the X and GX stages, respectively, also indicate active and inactive cAMP–PKA pathway status during the X and GX stages, respectively. Although the Snf3p/Rgt2p, Snf1–Mig1, and cAMP–PKA pathways are able to cross-communicate with each other, the cAMP–PKA pathway plays the most prominent role in responding to changes in glucose availability and initiates the signalling processes that promote cell growth, fermentative metabolism, and division [22, 23]. The activation of PKA in response to the presence of fermentable carbon sources is directed by intracellular levels of cAMP. A study investigating the short-term behaviour of cAMP signalling revealed that the cAMP concentration rapidly increases following glucose stimulation. After reaching a peak, the concentration of cAMP then declines to a new steady state that is higher than its initial concentration [66, 67]. Based on previous reports and our observations, we suggest that glucose-sensing systems become lethargic during the GX stage. For example, we hypothesized that the transfer of *S. cerevisiae* cells into medium containing only xylose as the carbon source would stimulate a cAMP peak followed by PKA activation. Although this response was not as high as the levels achieved when glucose was introduced (the specific xylose consumption rate was much lower than that of glucose), the “good” start built up fermentative metabolism in the cells and maintained their status in the X stage. In contrast, the cAMP peak was stimulated by glucose first during glucose–xylose co-fermentation, and then the cAMP concentration decreased to a level that was higher than the initial state. The remaining xylose in the medium did not represent a new environment for the cells, and therefore no new cAMP peak was stimulated. In this case, the cells remained on track to enter the lag phage.

To determine whether the down-regulated genes in EMP directly affect the post-glucose effect, we overexpressed *FBA1, TDH2,* and *GPM1* in BSGX001. Glyceraldehyde-3-phosphate is a substrate of *TDH2*, which encodes glyceraldehyde-3-phosphate dehydrogenase (GAPDH). Glyceraldehyde-3-phosphate is also a substrate of transaldolase and transketolase, which are important enzymes in the non-oxidative part of the PPP. Therefore, it is possible that a reduction in GAPDH activity resulting from the deletion of *TDH2* would increase the availability of glyceraldehyde-3-phosphate for transaldolase, thereby improving xylose fermentation by increasing PPP flux [68]. In contrast, overexpression of *TDH2* may decrease the availability of glyceraldehyde-3-phosphate for transaldolase and decrease xylose fermentation. Our result is consistent with this; overexpression of *TDH2* almost blocked the cell growth in the GX stage. Furthermore, overexpression of *FBA1* or *GPM1* did not affect xylose fermentation. This may be because the enzymes encoded by them are bidirectional and participate in both glycolysis and gluconeogenesis. The reaction direction may depend more on the substrate concentration than on the expression levels of these enzymes. Furthermore, several studies investigating the overexpression of glycolysis genes have suggested that glycolysis flux is not easily changed by altering individual enzyme activities [69].

Although the exact mechanism of the post-glucose effect remains unclear, we demonstrated that the disruption of related TFs mitigated this effect. In lieu of mining TFs from well-known regulatory networks that affect xylose metabolism [35], we investigated all TFs whose expression levels significantly differed between the GX and X stages for both test strains. Among these TFs, Adr1p, Znf1p, and Nrg1p, which are important for carbon metabolism, did not positively affect xylose metabolism, while Thi2p, Nrm1p, and Yhp1 unexpectedly did affect xylose metabolism. Thi2p is a transcriptional activator of thiamine biosynthetic genes that acts with Pdc2p to respond to thiamine diphosphate (TPP, also known as ThDP) demand; this TF is believed to be associated with carbon source availability [56]. ThDP is a cofactor for pyruvate dehydrogenase, pyruvate decarboxylase, and transketolase. Knockout of *THI2* led to relatively low pyruvate dehydrogenase complex and pyruvate decarboxylase activities, thus decreasing the cell growth rate and glucose metabolism [70]. However, the PPP is impaired by the down-regulation of ThDP-dependent transketolase due to *THI2* knockout [70]. Based on these results, knocking out *THI2* should exert a negative effect on xylose metabolism. Our results confirmed that knocking out *THI2* did, in fact, decrease growth and xylose utilization in the X stage. However, interestingly, the opposite result was observed in the GX stage. Knocking out *THI2* increased the specific growth rate, specific xylose utilization rate, and specific ethanol production rate of GX stage cells by 17.4, 26.8, and 32.4%, respectively (Table 5). Our analysis suggests that the enhanced expression of RPs caused by *THI2* deletion contributed to these increases. Furthermore, we observed that enhancing the expression of some RPs directly or by deleting *THI2* enhanced strain growth and xylose metabolism in the GX stage.

Overexpressing *NRM1* enhanced xylose fermentation not only in the GX stage, but also in the X stage, while deleting *NRM1* notably decreased xylose fermentation in the GX stage. Measurement of the proportion of cells in different cell cycle phases suggested that the cell cycle was affected by *NRM1* overexpression during the X stage. Furthermore, the specific growth rate of the *NRM1*-overexpressing strain was higher than that of the control. These results suggest that *NRM1* overexpression accelerated the cell cycle during the X stage. Moreover, the overexpression of *YHP1*, which encodes a different cell cycle-related TF, also enhanced cell growth in the X stage. However, specific xylose consumption was not affected by the overexpression of *YHP1*. Sfg1p and Swi5p are also important cell cycle TFs, but disrupting them did not benefit xylose fermentation. Therefore, we suggest that the regulation of *NRM1* (possibly via MBF) is more important than other regulatory factors in mediating the post-glucose effect in terms of the cell cycle. However, the exact mechanism remains unclear.

## Conclusion

The present study investigated the mechanisms underlying the post-glucose effect on xylose metabolism, a metabolic phenomenon commonly found in recombinant xylose-utilizing *S. cerevisiae*. Glucose-sensing systems become lethargic during glucose–xylose co-fermentation after glucose depletion; therefore, the cells do not respond to residual xylose and enter the lag phase. Knocking out *THI2* or overexpressing *NRM1* or *YHP1* increases xylose metabolism; such methods may be applied to alleviate the post-glucose effect and enhance xylose utilization. Their enhancement of xylose utilization is likely attributable to improved ribosome synthesis and alteration of the cell cycle.

## Additional file

**Additional file 1: Figure S1.** Fermentation characteristics of xylose-utilizing strains. **Figure S2.** The transcriptional difference of genes involved in fructose, mannose, galactose, sucrose, and starch metabolism in GX stage versus X stage in both BSGX001 and XH7. **Table S1.** The primers used in this study. **Table S2.** Gene cluster analysis of transcriptome difference of GX stage versus X stage in both BSGX001 and XH7. **Table S3.** Gene cluster analysis of transcription reaction in *THI2* deletion strains versus WT strains. **Table S4.** The ribosomal related genes with significantly different expression levels between GX stage and X stage in both BSGX001 and XH7.

## Abbreviations

TFs: transcription factors
XR: xylose reductase
XDH: xylitol dehydrogenase
XI: xylose isomerase
PPP: pentose phosphate pathway
LB: Luria–Bertani
SC-Ura: synthetic complete dropout uracil
SC: synthetic complete dropout
DCW: dry cell weight
GEO: Gene Expression Omnibus database
qPCR: quantitative polymerase chain reaction
GO: Gene Ontology
RPs: ribosomal proteins
MBF: MCB binding factor
ECB: early cell cycle box
GAPDH: glyceraldehyde-3-phosphate dehydrogenase
TPP (ThDP): thiamine diphosphate

## References

[1] Diao L, Liu Y, Qian F, Yang J, Jiang Y, Yang S (2013). Construction of fast xylose-fermenting yeast based on industrial ethanol-producing diploid *Saccharomyces cerevisiae* by rational design and adaptive evolution. BMC Biotechnol.

[2] Matsushika A, Nagashima A, Goshima T, Hoshino T (2013). Fermentation of xylose causes inefficient metabolic state due to carbon/energy starvation and reduced glycolytic flux in recombinant industrial *Saccharomyces cerevisiae*. PLoS ONE.

[3] Shen Y, Chen X, Peng B, Chen L, Hou J, Bao X (2012). An efficient xylose-fermenting recombinant *Saccharomyces cerevisiae* strain obtained through adaptive evolution and its global transcription profile. Appl Microbiol Biotechnol.

[4] Zhou H, Cheng JS, Wang BL, Fink GR, Stephanopoulos G (2012). Xylose isomerase overexpression along with engineering of the pentose phosphate pathway and evolutionary engineering enable rapid xylose utilization and ethanol production by *Saccharomyces cerevisiae*. Metab Eng.

[5] Kuyper M, Toirkens MJ, Diderich JA, Winkler AA, van Dijken JP, Pronk JT (2005). Evolutionary engineering of mixed-sugar utilization by a xylose-fermenting *Saccharomyces cerevisiae* strain. FEMS Yeast Res.

[6] Li H, Shen Y, Wu M, Hou J, Jiao C, Li Z, Liu X, Bao X (2016). Engineering a wild-type diploid *Saccharomyces cerevisiae* strain for second-generation bioethanol production. Bioresour Bioprocess.

[7] Sato TK, Tremaine M, Parreiras LS, Hebert AS, Myers KS, Higbee AJ, Sardi M, McIlwain SJ, Ong IM, Breuer RJ (2016). Correction: directed evolution reveals unexpected epistatic interactions that alter metabolic regulation and enable anaerobic xylose use by *Saccharomyces cerevisiae*. PLoS Genet.

[8] Bergdahl B, Heer D, Sauer U, Hahn-Hagerdal B, van Niel EW (2012). Dynamic metabolomics differentiates between carbon and energy starvation in recombinant *Saccharomyces cerevisiae* fermenting xylose. Biotechnol Biofuels.

[9] Bettiga M, Hahn-Hagerdal B, Gorwa-Grauslund MF (2008). Comparing the xylose reductase/xylitol dehydrogenase and xylose isomerase pathways in arabinose and xylose fermenting *Saccharomyces cerevisiae* strains. Biotechnol Biofuels.

[10] Kuyper M, Hartog MM, Toirkens MJ, Almering MJ, Winkler AA, van Dijken JP, Pronk JT (2005). Metabolic engineering of a xylose-isomerase-expressing *Saccharomyces cerevisiae* strain for rapid anaerobic xylose fermentation. FEMS Yeast Res.

[11] Johansson B, Hahn-Hagerdal B (2002). Overproduction of pentose phosphate pathway enzymes using a new CRE-loxP expression vector for repeated genomic integration in *Saccharomyces cerevisiae*. Yeast.

[12] Peng B, Shen Y, Li X, Chen X, Hou J, Bao X (2012). Improvement of xylose fermentation in respiratory-deficient xylose-fermenting *Saccharomyces cerevisiae*. Metab Eng.

[13] Toivari MH, Aristidou A, Ruohonen L, Penttila M (2001). Conversion of xylose to ethanol by recombinant *Saccharomyces cerevisiae*: importance of xylulokinase (XKS1) and oxygen availability. Metab Eng.

[14] Johansson B, Hahn-Hagerdal B (2002). The non-oxidative pentose phosphate pathway controls the fermentation rate of xylulose but not of xylose in *Saccharomyces cerevisiae* TMB3001. FEMS Yeast Res.

[15] Kim SR, Skerker JM, Kang W, Lesmana A, Wei N, Arkin AP, Jin YS (2013). Rational and evolutionary engineering approaches uncover a small set of genetic changes efficient for rapid xylose fermentation in *Saccharomyces cerevisiae*. PLoS ONE.

[16] Scalcinati G, Otero JM, Van Vleet JR, Jeffries TW, Olsson L, Nielsen J (2012). Evolutionary engineering of *Saccharomyces cerevisiae* for efficient aerobic xylose consumption. FEMS Yeast Res.

[17] Reider Apel A, Ouellet M, Szmidt-Middleton H, Keasling JD, Mukhopadhyay A (2016). Evolved hexose transporter enhances xylose uptake and glucose/xylose co-utilization in *Saccharomyces cerevisiae*. Sci Rep.

[18] Parreiras LS, Breuer RJ, Avanasi Narasimhan R, Higbee AJ, La Reau A, Tremaine M, Qin L, Willis LB, Bice BD, Bonfert BL (2014). Engineering and two-stage evolution of a lignocellulosic hydrolysate-tolerant *Saccharomyces cerevisiae* strain for anaerobic fermentation of xylose from AFEX pretreated corn stover. PLoS ONE.

[19] Hou J, Jiao C, Peng B, Shen Y, Bao X (2016). Mutation of a regulator Ask10p improves xylose isomerase activity through up-regulation of molecular chaperones in *Saccharomyces cerevisiae*. Metab Eng.

[20] Papini M, Nookaew I, Uhlen M, Nielsen J (2012). Scheffersomyces stipitis: a comparative systems biology study with the Crabtree positive yeast *Saccharomyces cerevisiae*. Microb Cell Fact.

[21] van Urk H, Postma E, Scheffers WA, van Dijken JP (1989). Glucose transport in crabtree-positive and crabtree-negative yeasts. J Gen Microbiol.

[22] Busti S, Coccetti P, Alberghina L, Vanoni M (2010). Glucose signaling-mediated coordination of cell growth and cell cycle in *Saccharomyces cerevisiae*. Sensors (Basel).

[23] Zaman S, Lippman SI, Schneper L, Slonim N, Broach JR (2009). Glucose regulates transcription in yeast through a network of signaling pathways. Mol Syst Biol.

[24] Ozcan S, Johnston M (1999). Function and regulation of yeast hexose transporters. Microbiol Mol Biol Rev.

[25] Gancedo JM (1998). Yeast carbon catabolite repression. Microbiol Mol Biol Rev.

[26] Dashko S, Zhou N, Compagno C, Piskur J (2014). Why, when, and how did yeast evolve alcoholic fermentation?. FEMS Yeast Res.

[27] Nissen TL, Hamann CW, Kielland-Brandt MC, Nielsen J, Villadsen J (2000). Anaerobic and aerobic batch cultivations of *Saccharomyces cerevisiae* mutants impaired in glycerol synthesis. Yeast.

[28] Zampar GG, Kummel A, Ewald J, Jol S, Niebel B, Picotti P, Aebersold R, Sauer U, Zamboni N, Heinemann M (2013). Temporal system-level organization of the switch from glycolytic to gluconeogenic operation in yeast. Mol Syst Biol.

[29] Salusjarvi L, Kankainen M, Soliymani R, Pitkanen JP, Penttila M, Ruohonen L (2008). Regulation of xylose metabolism in recombinant *Saccharomyces cerevisiae*. Microb Cell Fact.

[30] Salusjarvi L, Pitkanen JP, Aristidou A, Ruohonen L, Penttila M (2006). Transcription analysis of recombinant *Saccharomyces cerevisiae* reveals novel responses to xylose. Appl Biochem Biotechnol.

[31] Shen Y, Hou J, Bao X (2013). Enhanced xylose fermentation capacity related to an altered glucose sensing and repression network in a recombinant *Saccharomyces cerevisiae*. Bioengineered.

[32] Venkataram S, Dunn B, Li Y, Agarwala A, Chang J, Ebel ER, Geiler-Samerotte K, Herissant L, Blundell JR, Levy SF (2016). Development of a comprehensive genotype-to-fitness map of adaptation-driving mutations in yeast. Cell.

[33] Krahulec S, Petschacher B, Wallner M, Longus K, Klimacek M, Nidetzky B (2010). Fermentation of mixed glucose–xylose substrates by engineered strains of *Saccharomyces cerevisiae*: role of the coenzyme specificity of xylose reductase, and effect of glucose on xylose utilization. Microb Cell Fact.

[34] Demeke MM, Dietz H, Li Y, Foulquie-Moreno MR, Mutturi S, Deprez S, Den Abt T, Bonini BM, Liden G, Dumortier F (2013). Development of a d-xylose fermenting and inhibitor tolerant industrial *Saccharomyces cerevisiae* strain with high performance in lignocellulose hydrolysates using metabolic and evolutionary engineering. Biotechnol Biofuels.

[35] Michael DG, Maier EJ, Brown H, Gish SR, Fiore C, Brown RH, Brent MR (2016). Model-based transcriptome engineering promotes a fermentative transcriptional state in yeast. Proc Natl Acad Sci USA.

[36] Entian KD, Kötter P (1998). 23 Yeast mutant and plasmid collections.

[37] Li H, Wu M, Xu L, Hou J, Guo T, Bao X, Shen Y (2015). Evaluation of industrial *Saccharomyces cerevisiae* strains as the chassis cell for second-generation bioethanol production. Microb Biotechnol.

[38] Guldener U, Heck S, Fielder T, Beinhauer J, Hegemann JH (1996). A new efficient gene disruption cassette for repeated use in budding yeast. Nucleic Acids Res.

[39] Hou J, Shen Y, Jiao C, Ge R, Zhang X, Bao X (2016). Characterization and evolution of xylose isomerase screened from the bovine rumen metagenome in *Saccharomyces cerevisiae*. J Biosci Bioeng.

[40] Kogje AB, Ghosalkar A (2017). Xylitol production by genetically modified industrial strain of *Saccharomyces cerevisiae* using glycerol as co-substrate. J Ind Microbiol Biotechnol.

[41] Semkiv MV, Dmytruk KV, Sibirny AA (2016). Development of a system for multicopy gene integration in *Saccharomyces cerevisiae*. J Microbiol Methods.

[42] Sonderegger M, Jeppsson M, Hahn-Hagerdal B, Sauer U (2004). Molecular basis for anaerobic growth of *Saccharomyces cerevisiae* on xylose, investigated by global gene expression and metabolic flux analysis. Appl Environ Microbiol.

[43] Tarazona S, Garcia-Alcalde F, Dopazo J, Ferrer A, Conesa A (2011). Differential expression in RNA-seq: a matter of depth. Genome Res.

[44] Livak KJ, Schmittgen TD (2001). Analysis of relative gene expression data using real-time quantitative PCR and the 2(−Delta Delta C(T)) Method. Methods.

[45] Foss EJ (2001). Tof1p regulates DNA damage responses during S phase in *Saccharomyces cerevisiae*. Genetics.

[46] Van Vleet JH, Jeffries TW (2009). Yeast metabolic engineering for hemicellulosic ethanol production. Curr Opin Biotechnol.

[47] Macneil LT, Walhout AJ (2011). Gene regulatory networks and the role of robustness and stochasticity in the control of gene expression. Genome Res.

[48] Yu H, Gerstein M (2006). Genomic analysis of the hierarchical structure of regulatory networks. Proc Natl Acad Sci USA.

[49] Lin Y, Chomvong K, Acosta-Sampson L, Estrela R, Galazka JM, Kim SR, Jin YS, Cate JH (2014). Leveraging transcription factors to speed cellobiose fermentation by *Saccharomyces cerevisiae*. Biotechnol Biofuels.

[50] Young ET, Dombek KM, Tachibana C, Ideker T (2003). Multiple pathways are co-regulated by the protein kinase Snf1 and the transcription factors Adr1 and Cat8. J Biol Chem.

[51] Tangsombatvichit P, Semkiv MV, Sibirny AA, Jensen LT, Ratanakhanokchai K, Soontorngun N (2015). Zinc cluster protein Znf1, a novel transcription factor of non-fermentative metabolism in *Saccharomyces cerevisiae*. FEMS Yeast Res.

[52] Lee SB, Kang HS, Kim T (2013). Nrg1 functions as a global transcriptional repressor of glucose-repressed genes through its direct binding to the specific promoter regions. Biochem Biophys Res Commun.

[53] Garcia-Gimeno MA, Struhl K (2000). Aca1 and Aca2, ATF/CREB activators in *Saccharomyces cerevisiae*, are important for carbon source utilization but not the response to stress. Mol Cell Biol.

[54] Teng Y, Liu H, Gill HW, Yu Y, Waters R, Reed SH (2008). *Saccharomyces cerevisiae* Rad16 mediates ultraviolet-dependent histone H3 acetylation required for efficient global genome nucleotide-excision repair. EMBO Rep.

[55] Jansen ML, Daran-Lapujade P, de Winde JH, Piper MD, Pronk JT (2004). Prolonged maltose-limited cultivation of *Saccharomyces cerevisiae* selects for cells with improved maltose affinity and hypersensitivity. Appl Environ Microbiol.

[56] Mojzita D, Hohmann S (2006). Pdc2 coordinates expression of the THI regulon in the yeast *Saccharomyces cerevisiae*. Mol Genet Genomics.

[57] Ofir A, Hofmann K, Weindling E, Gildor T, Barker KS, Rogers PD, Kornitzer D (2012). Role of a *Candida albicans* Nrm1/Whi5 homologue in cell cycle gene expression and DNA replication stress response. Mol Microbiol.

[58] Pramila T, Miles S, GuhaThakurta D, Jemiolo D, Breeden LL (2002). Conserved homeodomain proteins interact with MADS box protein *Mcm*1 to restrict ECB-dependent transcription to the M/G1 phase of the cell cycle. Genes Dev.

[59] Moll T, Tebb G, Surana U, Robitsch H, Nasmyth K (1991). The role of phosphorylation and the CDC28 protein kinase in cell cycle-regulated nuclear import of the *S. cerevisiae* transcription factor SWI5. Cell.

[60] Cipollina C, van den Brink J, Daran-Lapujade P, Pronk JT, Porro D, de Winde JH (2008). *Saccharomyces cerevisiae* SFP1: at the crossroads of central metabolism and ribosome biogenesis. Microbiology.

[61] Hu Z, Killion PJ, Iyer VR (2007). Genetic reconstruction of a functional transcriptional regulatory network. Nat Genet.

[62] Matsushika A, Goshima T, Hoshino T (2014). Transcription analysis of recombinant industrial and laboratory *Saccharomyces cerevisiae* strains reveals the molecular basis for fermentation of glucose and xylose. Microb Cell Fact.

[63] Shamsuzzaman M, Bommakanti A, Zapinsky A, Rahman N, Pascual C, Lindahl L (2017). Analysis of cell cycle parameters during the transition from unhindered growth to ribosomal and translational stress conditions. PLoS ONE.

[64] Huberts DH, Niebel B, Heinemann M (2012). A flux-sensing mechanism could regulate the switch between respiration and fermentation. FEMS Yeast Res.

[65] Rodkaer SV, Faergeman NJ (2014). Glucose- and nitrogen sensing and regulatory mechanisms in *Saccharomyces cerevisiae*. FEMS Yeast Res.

[66] Gonzales K, Kayikci O, Schaeffer DG, Magwene PM (2013). Modeling mutant phenotypes and oscillatory dynamics in the *Saccharomyces cerevisiae* cAMP–PKA pathway. BMC Syst Biol.

[67] Ma P, Wera S, Van Dijck P, Thevelein JM (1999). The PDE1-encoded low-affinity phosphodiesterase in the yeast *Saccharomyces cerevisiae* has a specific function in controlling agonist-induced cAMP signaling. Mol Biol Cell.

[68] Linck A, Vu XK, Essl C, Hiesl C, Boles E, Oreb M (2014). On the role of GAPDH isoenzymes during pentose fermentation in engineered *Saccharomyces cerevisiae*. FEMS Yeast Res.

[69] Wang S, Spor A, Nidelet T, Montalent P, Dillmann C, de Vienne D, Sicard D (2011). Switch between life history strategies due to changes in glycolytic enzyme gene dosage in *Saccharomyces cerevisiae*. Appl Environ Microbiol.

[70] Xu G, Hua Q, Duan N, Liu L, Chen J (2012). Regulation of thiamine synthesis in *Saccharomyces cerevisiae* for improved pyruvate production. Yeast.

