# Supplementary material for: Disruption of the transcription factors Thi2p and Nrm1p alleviates the post-glucose effect on xylose utilization in Saccharomyces cerevisiae
Source: Biotechnol Biofuels. 2018 Apr 16;11:112. doi: 10.1186/s13068-018-1112-1 (PMC5901872; doi:10.1186/s13068-018-1112-1)
Supplement: Supplementary file 1 — Additional file 1: Figure S1. Fermentation characteristics of xylose-utilizing strains. Figure S2. The transcriptional difference of genes involved in fructose, mannose, galactose, sucrose, and starch metabolism in GX stage versus X stage in both BSGX001 and XH7. Table S1. The primers used in this study. Table S2. Gene cluster analysis of transcriptome difference of GX stage versus X stage in both BSGX001 and XH7. Table S3. Gene cluster analysis of transcription reaction in THI2 deletion strains versus WT strains. Table S4. The ribosomal related genes with significantly different expression levels between GX stage and X stage in both BSGX001 and XH7. [file 13068_2018_1112_MOESM1_ESM.doc]

**10 g L^-1^ xylose retained at sample time point**

**13 g L^-1^ xylose retained at sample time point**

**8 g L^-1^ xylose retained at sample time point**

**12 g L^-1^ xylose retained at sample time point**

Figure **S**1 Fermentation characteristics of xylose-utilizing strains. Cells were cultured in bioreactors at 30 °C and pH 5.5, with 0.06 vvm air sparging and a stirring speed of 200 rpm. Black arrow indicates ln (dry cell weight) in log scale; The red arrow indicates the residual xylose concentration at sample time point. (A), BSGX001, xylose fermentation; (B), BSGX001, glucose-xylose co-fermentation; (C), XH7, xylose fermentation; (D), XH7, glucose-xylose co-fermentation. Symbols:■, glucose; ●, xylose;▼, glycerol;◄ , acetate; ▶, ethanol; -, biomass. The red arrows indicate the time points that samples were taken for transcriptional analysis. All the data are the mean value of independent duplicate tests.


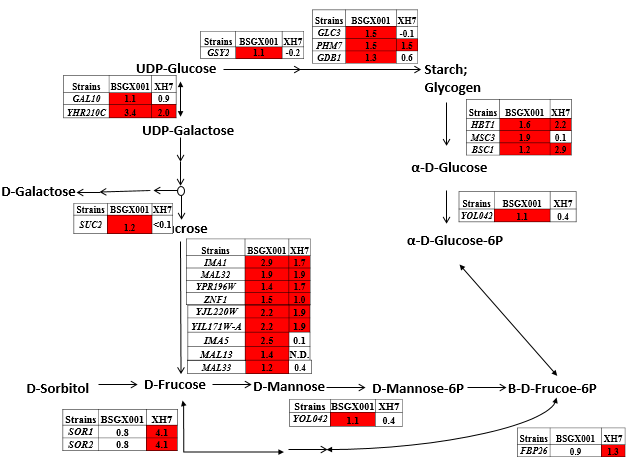


**Figure S2 The transcriptional difference of genes involved in fructose, mannose, galactose, sucrose, and starch metabolism in the GX stage versus the X stage in both BSGX001 and XH7.**

The indexes of significant differences of all the genes were a p-value of 0.001 or less, and an absolute fold-change threshold of 2.0 or greater.

**N.D.** represents not detected.

**Table S1 The primers used in this study.**

| Name | Sequence (5' → 3') |
| --- | --- |
| **Primers for Gene Deletion** | |
| *Kan(loxp)-F* | AGCTGAAGCTTCGTACGCTG |
| *Kan(loxp)-R* | CGACTCACTATAGGGAGACCG |
| *aca1-1-F* | AGATGGAAGGCAGAACCCAC |
| *aca1-1-R* | ATTAAGGGTTGTCGACCTGCAGCGTACGAAGCTTCAGCTGCGCCTTAGACGATAGGATC |
| *aca1-2-F* | TATGCGGCCGCGGATCTGCCGGTCTCCCTATAGTGAGTCGGCAGGCTTGAAAGACGGTG |
| *aca1-2-F* | CTGGAGCTGCGTATCATTTC |
| *adr1-1-F* | ACCAAACGATTGTTCAGGCTTTCCCGTTGTT |
| *adr1-1-R* | TGTCGACCTGCAGCGTACGAAGCTTCAGCTCAAGTGCTCTTGTCTTGCGAACGCTC |
| *adr1-2-F* | CGCGGATCTGCCGGTCTCCCTATAGTGAGTCGCCTTTGATAATGGGGACAGGGCAGTG |
| *adr1-2-F* | GTGTGGAAATCACTTGCCATCTTATCTAACATGTG |
| *znf1-1-F* | ATGGCCCGCAATAGACAAGCGTGC |
| *znf1-1-R* | ATTAAGGGTTGTCGACCTGCAGCGTACGAAGCTTCAGCTAGCGTGGCGGCAGAAAGAGATACC |
| *znf1-2-F* | TATGCGGCCGCGGATCTGCCGGTCTCCCTATAGTGAGTCGAGAACACTTGCTTGGAAGCTAGTGCTTC |
| *znf1-2-R* | GATTCAAGCGGATTAGAAAGAGGAAGCGTG |
| *rad16-1-F* | ATGCAAGAAGGGGGCTTTATCCG |
| *rad16-1-R* | ATTAAGGGTTGTCGACCTGCAGCGTACGAAGCTTCAGCTGTTAACGGTTCGTCGTCATCCG |
| *rad16-2-F* | TATGCGGCCGCGGATCTGCCGGTCTCCCTATAGTGAGTCGCTCCGTCTTTAGTTGTTGCCCCTAC |
| *rad16-2-R* | CCTTGATATTGTGTGCCTCATCCAG |
| *nrg1-1-F* | AATCTCCGCATTTGATGGAATG |
| *nrg1-1-R* | GCGTACGAAGCTTCAGCTTCCCACTTTTCAGAGCCATTTTC |
| *nrg1-2-F* | CCGGTCTCCCTATAGTGAGTCGCCGAAAAAAGCTCGCTATCAC |
| *nrg1-2-R* | GCTGCGTGCATCCCTTATAC |
| *ypr196w-1-F* | GACAGTCATGTGATTGCTGTCGCG |
| *ypr196w-1-R* | ATTAAGGGTTGTCGACCTGCAGCGTACGAAGCTTCAGCTGGCAGATCACTAAGAGTTGCTGCAG |
| *ypr196w-2-F* | GAAGTTATTAGGTGATATCAGATCCACTAGTGGCCTATGCCACTATATCTCCACCACAACCCG |
| *ypr196w-2-R* | GGAATGTGTGTGGCGTTAGTATTCG |
| *swi5-1-F* | GCTAGAGGCAGTGACCCTTCGAG |
| *swi5-1-R* | ATTAAGGGTTGTCGACCTGCAGCGTACGAAGCTTCAGCTCATCGTGGGGCTTATCACTC |
| *swi5-2-F* | TATGCGGCCGCGGATCTGCCGGTCTCCCTATAGTGAGTCGCGTCCAACTCTGCTAACATT |
| *swi5-2-R* | GCTCGGCGGCTTTAGATGTGGTG |
| *yhp1-1-F* | ATGGAAAGCAGAAATACCGTGC |
| *yhp1-1-R* | ATTAAGGGTTGTCGACCTGCAGCGTACGAAGCTTCAGCTGGGCTTGGATAAAGGCGTCA |
| *yhp1-2-F* | TATGCGGCCGCGGATCTGCCGGTCTCCCTATAGTGAGTCGCCTCTACGCCAACGAGTACG |
| *yhp1-2-R* | GAGATCGACCTCCCCAAGGGG |
| *thi2-1-F* | GTAGCGTCATCCTCCAAAGTGCC |
| *thi2-1-R* | GGGTTGTCGACCTGCAGCGTACGAAGCTTCAGCTTGTCGGGGGTGATAGTTGTC |
| *thi2-2-F* | GCGGATCTGCCGGTCTCCCTATAGTGAGTCGCGATGTATCCGCTGGTCAAC |
| *thi2-2-R* | CTAGTCCTGCATGGCATATACATCC |
| *RH(thi2)-F* | GACAACTATCACCCCCGACAAGCTGAAGCTTCGTACGCTG |
| *RH(thi2)-R* | GTTGACCAGCGGATACATCGCGACTCACTATAGGGAGACCG |
| *sfg1-1-F* | ATGGATGAAATGCATTCTTCAGAC |
| *sfg1-1-R* | CAGCGTACGAAGCTTCAGCTCTCAATCCAGGACTGACAATATTCC |
| *sfg1-2-F* | GCCGGTCTCCCTATAGTGAGTCGACCAGCAATTTCTAGTATTTGGG |
| *sfg1-2-R* | TTATTGTTCTAAAACCTTTGCCCAC |
| *RH(sfg1)-F* | TATTGTCAGTCCTGGATTGAGAGCTGAAGCTTCGTACGCTG |
| *RH(sfg1)-R* | AATACTAGAAATTGCTGGTCGACTCACTATAGGGAGACCG |
| *nrm1-1-F* | ATGTCCATTATGAAGCAGAGGCTAC |
| *nrm1-1-R* | CAGCGTACGAAGCTTCAGCTGCTCAGAGTAATCTCTAGACGTTG |
| *nrm1-2-F* | ATCTGCCGGTCTCCCTATAGTGAGTCGGCATAGCATTACGAGGCCTTCC |
| *nrm1-2-R* | GACTTAGCGGCTTTGATGCTTG |
| *RH(nrm1)-F* | CGTCTAGAGATTACTCTGAGCAGCTGAAGCTTCGTACGCTG |
| *RH(nrm1)-R* | GAAGGCCTCGTAATGCTATGCCGACTCACTATAGGGAGACCG |
| **Primers for Gene overexpression** | |
| *TPIp-F* | AGATCTACGTATGGTCATTTCTTC |
| *TPIp-R* | CTACAAAAAACACATACATAAACTAAAA |
| *TPIp-2-F* | GGTCTCCCTATAGTGAGTCGAGATCTACGTATGGTCATTTCTTC |
| *TPIp-1-R* | GAAGAAATGACCATACGTAGATCTCGACTCACTATAGGGAGACC |
| *ADR1-1-F* | GCTACTTTCCCGGTTCTCCCTTTATTATAAACACTTCAGAGCTGAAGCTTCGTACGCTGCAGGTC |
| *ADR1-2-R* | CGGGAAAGCCTGAACAATCGTTTGGTTTTTCTACGTTAGCCATTTTTAGTTTATGTATGTGTTTTTTGTAG |
| *ACA1-1-F* | GACGATTTACCCTGGTGGTACAGAAGATTATGTTACATAAAGCTGAAGCTTCGTACGCTGCAGGTC |
| *ACA1-2-R* | GAATCTGGACTTGTAGCAAAATTATGCTTATAGTCCATTTTTAGTTTATGTATGTGTTTTTTGTAG |
| *ZNF1-1-F* | ACTCCTGAGATCTGCCAAATCGGTAGCTTATTGAACTGCGAGCTGAAGCTTCGTACGCTGCAGGTC |
| *ZNF1-2-R* | GAATGCAACAACAGTCGCACGCTTGTCTATTGCGGGCCATTTTTAGTTTATGTATGTGTTTTTTGTAG |
| *RAD16-1-F* | CCGAGAAACCGTAACGGTTTCATGTTGACCGGTTTTTGAGTAGCTGAAGCTTCGTACGCTGC |
| *RAD16-2-R* | CACATACATAAACTAAAAATGCAAGAAGGGGGCTTTATCCGTAGAAGGCGTACGAGAAG |
| *NRG1-1-F* | GCTGCAGGCTCCTTTTCCGTTATTCATATATAAGGAAGAGAGCTGAAGCTTCGTACGCTGCAGGTC |
| *NRG1-2-R* | GTAGAAACATTGAGGTTACTATAGTTATATGGGTAAAACATTTTTAGTTTATGTATGTGTTTTTTGTAG |
| *YPR196W-1-F* | GGCTAATATCAAAGTATCCACGTATATCCCCATGACACCGAGCTGAAGCTTCGTACGCTGCAGGTC |
| *YPR196W-1-R* | CGAACGCGACAGCAATCACATGACTGTCTTACAATACTCATTTTTAGTTTATGTATGTGTTTTTTGTAG |
| *THI2-1-F* | TCTCGGTCGGAATTATATGGGGAACTAATACTACGGGTTAAGCTGAAGCTTCGTACGCTGCAGG |
| *THI2-2-R* | CTACTTTCTTGCTTCTCTGCTGCCTCTTACTATTGATCATTTTTAGTTTATGTATGTGTTTTTTG |
| *SWI5-1-F* | GCAGCATTGTTGGAAATATCTTGGTCTGAATCCCAATCCGAGCTGAAGCTTCGTACGCTGCAGGTC |
| *SWI5-2-R* | GTACTTTTGAGGCATCAAACCAAGAGTTTGATGTATCCATTTTTAGTTTATGTATGTGTTTTTTGTAG |
| *YHP1-1-F* | CCCGGAATGGTAAACATCAGACGCGCTTTCCGTGCAGGGAAGCTGAAGCTTCGTACGCTG |
| *YHP1-2-R* | TGTTTGGTAAAGAAGGAAGCACGGTATTTCTGCTTTCCATTTTTAGTTTATGTATGTGTT |
| *SFG1-1-F* | GATCGTAATTCGACACATAAAACTCTCAGCTAGCCCACCCAGCTGAAGCTTCGTACGCTGCAGG |
| *SFG1-2-R* | CGCAACAAAAGTGTGTCTGAAGAATGCATTTCATCCATTTTTAGTTTATGTATGTGTTTTTTG |
| *NRM1-1-F* | ATAGATTATATATACACAAACTTAAAACACTGTTTTCTAAAGCTGAAGCTTCGTACGCTGC |
| *NRM1-2-R* | CTCCCCCAGTGGTAGCCTCTGCTTCATAATGGACATTTTTAGTTTATGTATGTGTTTTTTGTAG |
| **Primers for Plasmid Construction** | |
| *FBA1-F* | GACCTTAATTAATTATAAAGTGTTAGTGGTACGG |
| *FBA1-R* | AAATATGCGGCCGCATGGGTGTTGAACAAATC |
| *TDH2-F* | GACCTTAATTAATTAAGCCTTGGCAACGTGTT |
| *TDH2-R* | AAATATGCGGCCGCATGGTTAGAGTTGCTATTAACGG |
| *GPM1-F* | GACCTTAATTAATTATTTCTTACCTTGGTTGGC |
| *GPM1-R* | AAATATGCGGCCGCATGCCAAAGTTAGTTTTAG |
| *RPL7A-F* | AAGGAAAAAAGCGGCCGCATGGCCGCTGAGTATGTATAC |
| *RPL7A-R* | CCTTAATTAATTAGTTCATGGACTTAACC |
| *RPL7B-F* | AAGGAAAAAAGCGGCCGCATGTCCACTGAGTATGTC |
| *RPL7B-R* | CCTTAATTAATTAGTTCATAGCCTTAACC |
| *RPL9A-F* | AAGGAAAAAAGCGGCCGCATGAAATACATCCAAACTGAAC |
| *RPL9A-R* | CCTTAATTAATTAATTATAAATCTTCAGTAATAAAAC |
| *RPL22A-F* | AAGGAAAAAAGCGGCCGCATGGCCCCAAACGTATGATAC |
| *RPL22A-R* | CCTTAATTAATTATTCTTCGTCAACAGTAATG |
| *RPL22B-1-F* | ATGGCTCCAAACACTTCCAGAAAGCAAAAAG |
| *RPL22B-1-R* | TTATTCGTCATCCTCTTCTTCGTCAGC |
| *RPL22B-2-F* | AAGGAAAAAAGCGGCCGCATGGCTCCAAACACTTCCAG |
| *RPL22B-2-R* | CCTTAATTAATTATTCGTCATCCTCTTCTTC |
| **Primers for quantitative PCR** | |
| *RPL9A-F* | TCAGCATCAAGTCCAGAATCGT |
| *RPL9A-R* | TCTGCCACCGTTGTGAACAG |
| *RPS9B- F* | AGCACATTGACTTCGCTCCA |
| *RPS9B- R* | TCTTCGTCTTCGGCTTCTTCA |
| *RPS9A- F* | TCGAAGGTAATGCTTTGATTAGGA |
| *RPS9A- R* | ACTCTTGCATGGTGGACAGAC |
| *RPS26B-F* | TGCCGTCAGAGATTTGTCCG |
| *RPS26B-R* | TGGCGTGAATAGCACAGGAG |
| *RPS22A-F* | CGCTGAAAAGACCGGTAAGC |
| *RPS22A-R* | TTCACCAATGTAACCGTGCT |
| *RPL8A-F* | TGGTTTCCACCATTGACGCT |
| *RPL8A-R* | GCTCTCTTGTCCATCTTGGCT |
| *RPL7A-F*  *RPL7A-R*  *RPL22A-F*  *RPL22A-R*  *RPL22B-F* | CTGAACAAGTCGCTGCTGAA  AGTAGGAACCAGCAGCCTTG  CCGTCGATGTCTCTTCTCCA  TGACAGTGACAGCGTTACCC  AACCGTTGACGTTTCCTCTCC |
| *RPL22B-R* | CCTCAATGGCGTTCCCTAAGTT |
| *RPL30-F* | CATCATTGCCGCTAACACTCC |
| *RPL30-R* | TCGTTGTTACCACCTTGGAAGT |

**Table S2 Gene cluster analysis of transcriptome difference of GX stage versus X stage in both BSGX001 and XH7.**

| **GO-Slim term (****Molecular Functions)** | **Cluster frequency** | **Genes annotated to the term** |
| --- | --- | --- |
| **Up-regulated** |  |  |
| transmembrane transporter activity | 11 out of 92 genes, 12% | *ADY2, AGP3, ALR2, ARN1, BIO5, CTR3, GIT1, JEN1, MAL31, PHO89, ZRT1* |
| hydrolase activity | 11 out of 92 genes, 12% | *ATH1, CLD1, CRR1, HEF3, IMA1, MAL32, PDE1, PRB1, RAD16, RSB1, UTR4* |
| transferase activity | 7 out of 92 genes, 7.6% | *BAT2, GPI18, NQM1, POT1, PRR2, RAD16, YPK2* |
| DNA binding | 6 out of 92 genes, 6.5% | *ACA1, ADR1, NRG1, RAD16, YPR196W, ZNF1* |
| nucleic acid binding transcription factor activity | 4 out of 92 genes, 4.3% | *ACA1, ADR1, NRG1, ZNF1* |
| oxidoreductase activity | 4 out of 92 genes, 4.3% | *GDH3, GPX1, PRX1, PUT1* |
| hydrolase activity, acting on glycosyl bonds | 3 out of 92 genes, 3.3% | *ATH1, IMA1, MAL32* |
| enzyme regulator activity | 3 out of 92 genes, 3.3% | *PAI3, REG2, SPL2* |
| ATPase activity | 3 out of 92 genes, 3.3% | *HEF3, RAD16, RSB1* |
| transcription factor binding | 2 out of 92 genes, 2.2% | *ADR1, NRG1* |
| RNA binding | 2 out of 92 genes, 2.2% | *HEF3, POT1* |
| kinase activity | 2 out of 92 genes, 2.2% | *PRR2, YPK2* |
| protein binding transcription factor activity | 2 out of 92 genes, 2.2% | *ADR1, NRG1* |
| lyase activity | 1 out of 92 genes, 1.1% | *PDC6* |
| translation factor activity, RNA binding | 1 out of 92 genes, 1.1% | *HEF3* |
| ligase activity | 1 out of 92 genes, 1.1% | *PYC1* |
| mRNA binding | 1 out of 92 genes, 1.1% | *POT1* |
| transferase activity, transferring glycosyl groups | 1 out of 92 genes, 1.1% | *GPI18* |
| peptidase activity | 1 out of 92 genes, 1.1% | *PRB1* |
| phosphatase activity | 1 out of 92 genes, 1.1% | *UTR4* |
| molecular function unknown | 46 out of 92 genes, 50% | *ATG36, BOP2, DIA1, DSF1, ECL1, FAT3, FMP33, FMP40, FMP46, GIS3, HBT1, JID1, MOH1, MPM1, NCA3, RGI2, RRT1, SPG4, SPO73, SUE1, TMA10, UGX2, ULI1, YBR051W, YCL012C, YDR461C-A, YET2, YFL051C, YGL165C, YGR201C, YHR210C, YIL059C, YIL171W-A, YJL144W, YJL220W, YJR115W, YML131W, YMR114C, YMR181C, YNL013C, YNL195C, YNR014W, YOR389W, YPL277C, YPR150W, ZPS1* |
| can not be mapped to a GO slim term | 4 out of 92 genes, 4.3% | *ATG8, CYC7, PEX18, THI73* |
| **Down-regulated** |  |  |
| structural constituent of ribosome | 10 out of 43 genes, 23.3% | *RPL22A, RPL22B, RPL7A, RPL7B, RPL8A, RPL9A, RPS22A, RPS26B,RPS9A, RPS9B* |
| transferase activity | 10 out of 43 genes, 23.3% | *ARO8, ELO2, HIS1, LEU9, MET1, MET14, MET3, MET6, TKL1, UTR2* |
| oxidoreductase activity | 6 out of 43 genes, 14% | *ALD5, LYS9, MET16, MET5, SER3, TDH2* |
| lyase activity | 4 out of 43 genes, 9.3% | *ACO2, FBA1, LEU1, LYS4* |
| DNA binding | 3 out of 43 genes, 7% | *HTA2, HTB2, SWI5* |
| methyltransferase activity | 2 out of 43 genes, 4.7% | *MET1, MET6* |
| transferase activity, transferring glycosyl groups | 2 out of 43 genes, 4.7% | *HIS1, UTR2* |
| transmembrane transporter activity | 2 out of 43 genes, 4.7% | *CTP1, GGC1* |
| rRNA binding | 1 out of 43 genes, 2.3% | *RPS9A* |
| nucleic acid binding transcription factor activity | 1 out of 43 genes, 2.3% | *SWI5* |
| ligase activity | 1 out of 43 genes, 2.3% | *ASN1* |
| nucleotidyltransferase activity | 1 out of 43 genes, 2.3% | *MET3* |
| enzyme regulator activity | 1 out of 43 genes, 2.3% | *CLB2* |
| RNA binding | 1 out of 43 genes, 2.3% | *RPS9A* |
| protein binding transcription factor activity | 1 out of 43 genes, 2.3% | *SWI5* |
| kinase activity | 1 out of 43 genes, 2.3% | *MET14* |
| molecular function unknown | 6 out of 43 genes, 14% | *AIM20, BUD28, SFG1, SRL1, YOR108C-A, YPL197C* |
| **GO-Slim term(Biological Processes)** | **Cluster frequency** | **Genes annotated to the term** |
| **Up-regulated** |  |  |
| response to chemical | 11 out of 92 genes, 12% | *ADR1, ATH1, GPX1, HBT1, NQM1, NRG1, PRR2, PRX1, ULI1, YJL144W, ZNF1* |
| ion transport | 10 out of 92 genes, 10.9% | *ADY2, AGP3, ALR2, CTR3, FAT3, GIT1, JEN1, PHO89, RSB1, ZRT1* |
| transmembrane transport | 8 out of 92 genes, 8.7% | *ADY2, AGP3, ALR2, GIT1, JEN1, PEX18, PHO89, ZRT1* |
| transcription from RNA polymerase II promoter | 6 out of 92 genes, 6.5% | *ACA1, ADR1, NRG1, PRR2, REG2, ZNF1* |
| cellular amino acid metabolic process | 5 out of 92 genes, 5.4% | *BAT2, GDH3, PDC6, PUT1, UTR4* |
| carbohydrate metabolic process | 4 out of 92 genes, 4.3% | *ATH1, IMA1, MAL32, PYC1* |
| lipid metabolic process | 4 out of 92 genes, 4.3% | *ADR1, CLD1, GPI18, POT1* |
| peroxisome organization | 4 out of 92 genes, 4.3% | *ADR1, ATG36, GPX1, PEX18* |
| sporulation | 3 out of 92 genes, 3.3% | *CRR1, PRB1, SPO73* |
| monocarboxylic acid metabolic process | 3 out of 92 genes, 3.3% | *ADR1, BIO5, POT1* |
| oligosaccharide metabolic process | 3 out of 92 genes, 3.3% | *ATH1, IMA1, MAL32* |
| protein targeting | 3 out of 92 genes, 3.3% | *ATG8, PEX18, SPL2* |
| response to oxidative stress | 3 out of 92 genes, 3.3% | *GPX1, NQM1, PRX1* |
| signaling | 3 out of 92 genes, 3.3% | *GIS3, PDE1, ULI1* |
| response to starvation | 3 out of 92 genes, 3.3% | *ATG36, ATG8, PRB1* |
| cell wall organization or biogenesis | 2 out of 92 genes, 2.2% | *CRR1, SPO73* |
| generation of precursor metabolites and energy | 2 out of 92 genes, 2.2% | *CYC7, RGI2* |
| mitochondrion organization | 2 out of 92 genes, 2.2% | *ATG8, NCA3* |
| lipid transport | 2 out of 92 genes, 2.2% | *FAT3, RSB1* |
| conjugation | 2 out of 92 genes, 2.2% | *HBT1, PRR2* |
| pseudohyphal growth | 2 out of 92 genes, 2.2% | *DIA1, NRG1* |
| protein phosphorylation | 2 out of 92 genes, 2.2% | *PRR2, YPK2* |
| invasive growth in response to glucose limitation | 2 out of 92 genes, 2.2% | *DIA1, NRG1* |
| meiotic cell cycle | 2 out of 92 genes, 2.2% | *CRR1, SPO73* |
| cellular respiration | 1 out of 92 genes, 1.1% | *CYC7* |
| cellular response to DNA damage stimulus | 1 out of 92 genes, 1.1% | *RAD16* |
| vacuole organization | 1 out of 92 genes, 1.1% | *ATG8* |
| translational elongation | 1 out of 92 genes, 1.1% | *HEF3* |
| regulation of organelle organization | 1 out of 92 genes, 1.1% | *ADR1* |
| protein lipidation | 1 out of 92 genes, 1.1% | *GPI18* |
| DNA repair | 1 out of 92 genes, 1.1% | *RAD16* |
| proteolysis involved in cellular protein catabolic process | 1 out of 92 genes, 1.1% | *RAD16* |
| chromatin organization | 1 out of 92 genes, 1.1% | *ADR1* |
| cofactor metabolic process | 1 out of 92 genes, 1.1% | *BIO5* |
| cell morphogenesis | 1 out of 92 genes, 1.1% | *HBT1* |
| membrane invagination | 1 out of 92 genes, 1.1% | *ATG8* |
| nucleobase-containing small molecule metabolic process | 1 out of 92 genes, 1.1% | *CYC7* |
| Golgi vesicle transport | 1 out of 92 genes, 1.1% | *ATG8* |
| organelle assembly | 1 out of 92 genes, 1.1% | *ATG8* |
| vitamin metabolic process | 1 out of 92 genes, 1.1% | *BIO5* |
| protein modification by small protein conjugation or removal | 1 out of 92 genes, 1.1% | *RAD16* |
| amino acid transport | 1 out of 92 genes, 1.1% | *AGP3* |
| response to osmotic stress | 1 out of 92 genes, 1.1% | *NRG1* |
| membrane fusion | 1 out of 92 genes, 1.1% | *ATG8* |
| biological process unknown | 35 out of 92 genes, 38.0% | *BOP2, DSF1, ECL1, FMP33, FMP40, FMP46, JID1, MOH1, MPM1, RRT1, SPG4, TMA10, UGX2, YBR051W, YCL012C, YDR461C-A, YET2, YFL051C, YGL165C, YGR201C, YHR210C, YIL059C, YIL171W-A, YJL220W, YJR115W, YML131W, YMR114C, YMR181C, YNL013C, YNL195C, YNR014W, YOR389W, YPL277C, YPR150W, ZPS1* |
| can not be mapped to a GO slim term | 6 out of 92 genes, 6.5% | *ARN1, MAL31, PAI3, SUE1, THI73, YPR196W* |
| **Down-regulated** |  |  |
| cellular amino acid metabolic process | 14 out of 43 genes, 32.6% | *ARO8, ASN1, HIS1, LEU1, LEU9, LYS4, LYS9, MET1, MET14, MET16, MET3, MET5, MET6, SER3* |
| cytoplasmic translation | 9 out of 43 genes, 20.9% | *RPL22A, RPL22B, RPL7A, RPL7B, RPL8A, RPL9A, RPS22A, RPS26B, RPS9A* |
| rRNA processing | 5 out of 43 genes, 11.6% | *RPL7A, RPL7B, RPL8A, RPS9A, RPS9B* |
| monocarboxylic acid metabolic process | 4 out of 43 genes, 9.3% | *ALD5, ELO2, FBA1, TDH2* |
| mitotic cell cycle | 4 out of 43 genes, 9.3% | *BUD28, CLB2, SFG1, SWI5* |
| cofactor metabolic process | 4 out of 43 genes, 9.3% | *FBA1, MET1, TDH2, TKL1* |
| biological process unknown | 4 out of 43 genes, 9.3% | *ACO2, AIM20, YOR108C-A, YPL197C* |
| carbohydrate metabolic process | 3 out of 43 genes, 7% | *FBA1, TDH2, UTR2* |
| nucleobase-containing small molecule metabolic process | 3 out of 43 genes, 7% | *FBA1, TDH2, TKL1* |
| ribosomal large subunit biogenesis | 3 out of 43 genes, 7% | *RPL7A, RPL7B, RPL8A* |
| cell wall organization or biogenesis | 2 out of 43 genes, 4.7% | *SRL1, UTR2* |
| generation of precursor metabolites and energy | 2 out of 43 genes, 4.7% | *FBA1, TDH2* |
| nucleobase-containing compound transport | 2 out of 43 genes, 4.7% | *GGC1, RPS26B* |
| ribosomal small subunit biogenesis | 2 out of 43 genes, 4.7% | *RPS9A, RPS9B* |
| transmembrane transport | 2 out of 43 genes, 4.7% | *CTP1, GGC1* |
| chromatin organization | 2 out of 43 genes, 4.7% | *HTA2, HTB2* |
| cytoskeleton organization | 1 out of 43 genes, 2.3% | *CLB2* |
| cytokinesis | 1 out of 43 genes, 2.3% | *BUD28* |
| cellular response to DNA damage stimulus | 1 out of 43 genes, 2.3% | *HTA2* |
| regulation of cell cycle | 1 out of 43 genes, 2.3% | *CLB2* |
| regulation of DNA metabolic process | 1 out of 43 genes, 2.3% | *SWI5* |
| translational elongation | 1 out of 43 genes, 2.3% | *RPS9B* |
| mitochondrion organization | 1 out of 43 genes, 2.3% | *GGC1* |
| regulation of organelle organization | 1 out of 43 genes, 2.3% | *CLB2* |
| DNA repair | 1 out of 43 genes, 2.3% | *HTA2* |
| transcription from RNA polymerase II promoter | 1 out of 43 genes, 2.3% | *SWI5* |
| regulation of protein modification process | 1 out of 43 genes, 2.3% | *CLB2* |
| ion transport | 1 out of 43 genes, 2.3% | *CTP1* |
| pseudohyphal growth | 1 out of 43 genes, 2.3% | *SFG1* |
| protein phosphorylation | 1 out of 43 genes, 2.3% | *CLB2* |
| protein dephosphorylation | 1 out of 43 genes, 2.3% | *CLB2* |
| lipid metabolic process | 1 out of 43 genes, 2.3% | *ELO2* |
| nuclear transport | 1 out of 43 genes, 2.3% | *RPS26B* |
| organelle fission | 1 out of 43 genes, 2.3% | *SWI5* |
| cellular ion homeostasis | 1 out of 43 genes, 2.3% | *GGC1* |
| regulation of translation | 1 out of 43 genes, 2.3% | *RPS9B* |

| **Table S3 Gene cluster analysis of transcription reaction in *THI2* deletion strains versus WT strains.** | | |
| --- | --- | --- |
| **GO-Slim term** | **Cluster frequency** | **Genes annotated to the term** |
| **Up-regulated**  structural constituent of ribosome | 66 out of 176 genes, 37.5% | *MRP17, MRPL33, NAM9, RDN25-1, RPL11B, RPL12B, RPL14A, RPL14B, RPL16A, RPL19A, RPL19B, RPL20B, RPL22A, RPL22B, RPL23B, RPL24A, RPL24B, RPL25, RPL26A, RPL26B, RPL27B, RPL28, RPL29, RPL30, RPL31A, RPL32, RPL33A, RPL33B, RPL35A, RPL35B, RPL36A, RPL36B, RPL37A, RPL37B, RPL38, RPL40A, RPL40B, RPL41A, RPL41B, RPL42A, RPL42B, RPL43A, RPL43B, RPP1A, RPS10B, RPS12, RPS15, RPS16B, RPS17A, RPS17B, RPS19A, RPS19B, RPS21A, RPS21B, RPS22B, RPS23A, RPS25A, RPS25B, RPS26B, RPS27A, RPS28A, RPS29A, RPS29B, RPS30A, RPS30B, RPS31* |
| structural molecule activity | 66 out of 176 genes, 37.5% | *MRP17, MRPL33, NAM9, RDN25-1, RPL11B, RPL12B, RPL14A, RPL14B, RPL16A, RPL19A, RPL19B, RPL20B, RPL22A, RPL22B, RPL23B, RPL24A, RPL24B, RPL25, RPL26A, RPL26B, RPL27B, RPL28, RPL29, RPL30, RPL31A, RPL32, RPL33A, RPL33B, RPL35A, RPL35B, RPL36A, RPL36B, RPL37A, RPL37B, RPL38, RPL40A, RPL40B, RPL41A, RPL41B, RPL42A, RPL42B, RPL43A, RPL43B, RPP1A, RPS10B, RPS12, RPS15, RPS16B, RPS17A, RPS17B, RPS19A, RPS19B, RPS21A, RPS21B, RPS22B, RPS23A, RPS25A, RPS25B, RPS26B, RPS27A, RPS28A, RPS29A, RPS29B, RPS30A, RPS30B, RPS31* |
| molecular function unknown | 42 out of 176 genes, 23.9% | *APQ12, BUD19, BUD28, EMC6, FMP27, FMP32, GOT1, HMF1, LCL2, MAK11, MIC12, MIM1, OPI11, PGA2, PHM6, PML1, PML39, POC4, SDD2, SET6, SRB6, TMA7, TSR2, TVP18, YAL045C, YBL028C, YBR090C, YCL007C, YDL241W, YDR209C, YJL152W, YJR085C, YLR162W, YLR297W, YML094C-A, YMR085W, YNL150W, YPL225W, YPR053C, YPR172W, YSY6, ZEO1* |
| RNA binding | 20 out of 176 genes, 11.4% | *LSM2, LSM5, LSM6, NOP10, RPL14A, RPL14B, RPL16A, RPL24A, RPL24B, RPL25, RPL26A, RPL26B, RPL28, RPL30, RPL36A, RPL36B, SNR44, SNU13, SUI1, TIF11* |
| transmembrane transporter activity | 11 out of 176 genes, 6.3% | *ATP15, ATP17, ATP18, ATP19, ATP20, CAN1, COX7, COX8, PHO89, TOM6, TOM7* |
| oxidoreductase activity | 9 out of 176 genes, 5.1% | *COX7, COX8, CUP1-2, ERV2, GPX2, GRX2, SOD1, SPS19, TRX1* |
| DNA binding | 9 out of 176 genes, 5.1% | *HHF1, HHF2, HMLALPHA1, HTA1, HTA2, HTB2, NHP6A, NRG2, RIM1* |
| hydrolase activity | 8 out of 176 genes, 4.5% | *ATP15, ATP17, ATP18, ATP19, ATP20, DUT1, FCY1, SPC2* |
| transferase activity | 8 out of 176 genes, 4.5% | *GRX2, OST4, RDL1, RPA12, RPB10, RPB11, RPB5, RPC10* |
| ion binding | 6 out of 176 genes, 3.4% | *ACB1, CMD1, CUP1-2, PMP3, RPB10, RPC10* |
| nucleotidyltransferase activity | 5 out of 176 genes, 2.8% | *RPA12, RPB10, RPB11, RPB5, RPC10* |
| ATPase activity | 5 out of 176 genes, 2.8% | *ATP15, ATP17, ATP18, ATP19, ATP20* |
| isomerase activity | 4 out of 176 genes, 2.3% | *ERV2, FPR1, RIB4, TOP3* |
| protein transporter activity | 4 out of 176 genes, 2.3% | *TIM10, TIM8, TOM6, TOM7* |
| unfolded protein binding | 3 out of 176 genes, 1.7% | *EGD1, HSP10, TIM10* |
| enzyme binding | 2 out of 176 genes, 1.1% | *NTF2, SNA3* |
| translation factor activity, RNA binding | 2 out of 176 genes, 1.1% | *SUI1, TIF11* |
| chromatin binding | 2 out of 176 genes, 1.1% | *NHP6A, REC8* |
| guanyl-nucleotide exchange factor activity | 2 out of 176 genes, 1.1% | *SBH2, SDO1* |
| enzyme regulator activity | 2 out of 176 genes, 1.1% | *RPP1A, SPL2* |
| protein binding, bridging | 1 out of 176 genes, 0.6% | *OST4* |
| rRNA binding | 1 out of 176 genes, 0.6% | *SNR44* |
| RNA modification guide activity | 1 out of 176 genes, 0.6% | *SNR44* |
| lipid binding | 1 out of 176 genes, 0.6% | *PMP3* |
| lyase activity | 1 out of 176 genes, 0.6% | *HEM15* |
| transferase activity, transferring glycosyl groups | 1 out of 176 genes, 0.6% | *OST4* |
| peptidase activity | 1 out of 176 genes, 0.6% | *SPC2* |
| transcription factor activity | 1 out of 176 genes, 0.6% | *HMLALPHA1* |
| can not be mapped to a GO slim Term |  | *DAP1, SAG1, SMT3, SNC2, SNL1, URM1* |
| **Down-regulated** |  |  |
| molecular function unknown | 37 out of 139 genes, 26.6% | *AIM17, APL1, BSC3, CIS1, CMC2, CSS1, DEF1, ERP2, FIT2, FIT3, FRA1, FYV8, MIT1, NPR3, OPI6, PFS1, PRM5, RTS3, SDS23, SPT21, TAO3, YAL004W, YAR064W, YAR068W, YCR013C, YDL206W, YEL075C, YEL076C-A, YFL067W, YHL049C, YHR214W, YKL177W, YLR217W, YLR463C, YLR464W, YPL182C, YPR203W* |
| RNA binding | 33 out of 139 genes, 23.7% | *NPL3, PRP24, SLH1, SSA1, TDH3, UTP20, YAR009C, YAR010C, YBL005W-B, YBR012W-A, YBR012W-B, YCL020W, YDR034C-C, YDR034C-D, YDR098C-A, YDR098C-B, YDR210C-C, YDR210C-D, YDR261C-C, YDR316W-B, YFL002W-A, YGR038C-A, YGR161W-A, YHR214C-C, YJR027W, YJR029W, YLR035C-A, YMR045C, YMR046C, YMR050C, YMR051C, YOL103W-B, YOR192C-B* |
| hydrolase activity | 31 out of 139 genes, 22.3% | *ATP6, DSE2, DUG1, ESP1, HSP82, MEF2, PMA1, PPH3, PTP1, RSC8, SLH1, SSA1, SSC1, TSL1, YAR009C, YBL005W-B, YBL113C, YBR012W-B, YDR034C-D, YDR098C-B, YDR210C-D, YDR316W-B, YFL002W-A, YJR027W, YJR029W, YLL066C, YMR045C, YMR050C, YOL103W-B, YOR192C-B, YRF1-5* |
| transferase activity | 27 out of 139 genes, 19.4% | *ARO8, BAT2, CAR2, CDC19, DMA1, LYS21, MCT1, PGK1, PMT1, PMT4, PRI2, TMT1, TSL1, YAR009C, YBL005W-B, YBR012W-B, YDR034C-D, YDR098C-B, YDR210C-D, YDR316W-B, YFL002W-A, YJR027W, YJR029W, YMR045C, YMR050C, YOL103W-B, YOR192C-B* |
| peptidase activity | 16 out of 139 genes, 11.5% | *DUG1, ESP1, YAR009C, YBL005W-B, YBR012W-B, YDR034C-D, YDR098C-B, YDR210C-D, YDR316W-B, YFL002W-A, YJR027W, YJR029W, YMR045C, YMR050C, YOL103W-B, YOR192C-B* |
| nucleotidyltransferase activity | 15 out of 139 genes, 10.8% | *PRI2, YAR009C, YBL005W-B, YBR012W-B, YDR034C-D, YDR098C-B, YDR210C-D, YDR316W-B, YFL002W-A, YJR027W, YJR029W, YMR045C, YMR050C, YOL103W-B, YOR192C-B* |
| nuclease activity | 14 out of 139 genes, 10.1% | *YAR009C, YBL005W-B, YBR012W-B, YDR034C-D, YDR098C-B, YDR210C-D, YDR316W-B, YFL002W-A, YJR027W, YJR029W, YMR045C, YMR050C, YOL103W-B, YOR192C-B* |
| transmembrane transporter activity | 10 out of 139 genes, 7.2% | *ATP6, COX15, ENB1, FET3, FTR1, MMP1, NRT1, ORT1, PMA1, SIT1* |
| oxidoreductase activity | 9 out of 139 genes, 6.5% | *ALD6, COX15, ERG3, ERG4, FET3, FRE1, FRE4, TDH1, TDH3* |
| lyase activity | 7 out of 139 genes, 5.0% | *ACO1, ENO1, ENO2, ERR2, GLY1, IRC7, PDC5* |
| DNA binding | 7 out of 139 genes, 5.0% | *ACO1, CHA4, CRZ1, MIF2, PHO2, PRI2, RSC8* |
| ATPase activity | 6 out of 139 genes, 4.3% | *ATP6, HSP82, PMA1, RSC8, SSA1, SSC1* |
| nucleic acid binding transcription factor activity | 4 out of 139 genes, 2.9% | *CHA4, CRZ1, MSS11, PHO2* |
| unfolded protein binding | 4 out of 139 genes, 2.9% | *CCT6, HSP42, HSP82, SSA1* |
| helicase activity | 4 out of 139 genes, 2.9% | *SLH1, YBL113C, YLL066C, YRF1-5* |
| enzyme regulator activity | 4 out of 139 genes, 2.9% | *BUD14, SSC1, SSE2, TSL1* |
| mRNA binding | 3 out of 139 genes, 2.2% | *NPL3, SLH1, UTP20* |
| transferase activity, transferring glycosyl groups | 3 out of 139 genes, 2.2% | *PMT1, PMT4, TSL1* |
| phosphatase activity | 3 out of 139 genes, 2.2% | *PPH3, PTP1, TSL1* |
| protein binding, bridging | 1 out of 139 genes, 0.7% | *SLA2* |
| enzyme binding | 1 out of 139 genes, 0.7% | *NPL3* |
| methyltransferase activity | 1 out of 139 genes, 0.7% | *TMT1* |
| hydrolase activity, acting on glycosyl bonds | 1 out of 139 genes, 0.7% | *DSE2* |

**Table S4 The ribosomal related genes with significantly different expression levels between GX stage and X stage in both BSGX001 and XH7.**

| Genotype | Log_2_Ratio(GX/X) | |
| --- | --- | --- |
|  | BSGX001 | XH7 |
| *RPL7A /// RPL7B* | -1.145 | -1.430 |
| *RPL8A /// RPL8B* | -1.682 | -1.355 |
| *RPL9A* | -1.183 | -1.040 |
| *RPL22A* | -1.177 | -1.417 |
| *RPL22B* | -1.298 | -2.007 |
| *RPS9A* | -1.306 | -1.027 |
| *RPS9B* | -1.011 | -1.130 |
| *RPS22A* | -1.400 | -1.124 |
| *RPS26B* | -1.345 | -1.582 |
